# Application of 3D-printed compensators for proton pencil beam scanning of shallowly localized pediatric tumors

**DOI:** 10.1186/s13014-025-02646-3

**Published:** 2025-04-29

**Authors:** Agnieszka Wochnik, Tomasz Kajdrowicz, Gabriela Foltyńska, Dawid Krzempek, Katarzyna Krzempek, Krzysztof Małecki, Marzena Rydygier, Jan Swakoń, Paweł Olko, Renata Kopeć

**Affiliations:** 1 Institute of Nuclear Physics, Polish Academy of Sciences, Krakow, Poland; 2 University Children’s Hospital of Krakow, Krakow, Poland

**Keywords:** Proton radiotherapy, Pencil beam scanning, Pediatric tumors, Range discriminators, Additive technology, 3D-printed beam compensators

## Abstract

**Background:**

In modern proton radiotherapy facilities with pencil beam scanning technology, the lowest energy of a proton beam typically ranges between 60 and 100 MeV, corresponding to a proton range in water of 3.1–7.5 cm. The irradiation of superficial lesions usually requires the application of a range shifter (RS) to further reduce the proton range. A certain distance from the patient to the RS increases the spot size, causing worse plan conformity. As an alternative solution, a patient-specific 3D-printed proton beam compensator (BC) can be applied to reduce the air gap and beam scattering.

**Materials and methods:**

This study is based on treatment planning system simulations using retrospectively selected data from six pediatric patients with diagnosed sarcomas located in the head and neck area. For three of these patients, 3D-printed compensators were utilized during the treatment phase, prior to the retrospective analysis. Treatment plans for children with shallow lesions treated using RSs and BCs were compared. Planning target volume constraints (D_98%_ >95%, D_2%_< 107%) and organs-at-risk (brainstem, spinal cord, visual organs, chiasm, cochlea) constraints (D_2%_, D_max_ and D_Mean_) were applied. The entire process of using a BCs in the treatment of pediatric superficial tumors is presented, including 3D printing procedure (via fused filament fabrication method), dosimetric verification of the material (Water Equivalent Ratio measurements) and assessment of its homogeneity, print quality and Hounsfield Unit specification. Beam parameters analysis including spot sizes and penumbras, were performed. Treatment plans were compared in terms of plan conformity and sparing of critical organs.

**Results:**

The application of BCs reduced the low-dose irradiation areas, improved conformity and reduced critical organs exposure. BCs decreased the lateral spot size by approximately 57% and the penumbras by 41–47% at different depths in the cube target. The variation in BC homogeneity was less than 3.5%, meeting the criteria for plan robustness evaluation.

**Conclusions:**

Compared with RS placement at the nozzle, the placement of 3D-printed BCs in the near vicinity of the patient for the treatment of superficial tumors led to a more conformal dose distribution.

**Supplementary Information:**

The online version contains supplementary material available at 10.1186/s13014-025-02646-3.

## Background

Proton therapy (PT) is an advanced form of radiotherapy increasingly employed for treating various cancer [1, 2]. The application of proton beam is characterized by very beneficial physical properties which contribute to the excellent dose distribution in the patient. PT significantly reduces the risk of damage to healthy organs surrounding the lesion, which may decrease radiation-induced adverse effects [3]. These properties are particularly important for pediatric patient treatment. Treatment-related toxicities may contribute to the occurrence of secondary tumors (stochastic effects), as well as a reduction in irradiated organ function or growth retardation (deterministic effects) [4, 5]. Compared to conventional photon-based radiotherapy, PT achieves substantially lower total doses to healthy tissues [3].

The most conformal proton treatment plans can be obtained using the pencil beam scanning (PBS) technique [2]. Compared to passive-scattering proton systems, the application of the PBS technique enables more conformal dose distributions for the treated volume while sparing more healthy tissue and reducing neutron doses [6]. In proton therapy facilities with PBS technology, the lowest energy of the proton beam typically ranges from 60 to 100 MeV, which corresponds to a range of approximately 3.1–7.5 cm in water-equivalent thickness (WET) [6]. The irradiation of superficial lesions requires the application of preabsorbing range shifters (RSs) to deliver more shallow spots [7, 8]. RSs, typically fixed and attached to the nozzle at a certain distance from the patient, increase beam scattering, leading to an increased lateral spot size, beam penumbra and compromised conformity [9, 10]. To minimize these effects, some facilities use movable RSs that can reduce the air gap and improve scattering parameters [8, 10, 11], but their use require careful treatment planning to prevent collisions. Centers without movable RSs or table adjustments face challenges in treating shallow tumors due to the limitations of fixed RSs.

Efforts to overcome these issues led to the development of the so-called universal bolus (UB) as a substitute for the RS [12]. Designed for head and neck tumor treatments, the UB featured a consistent thickness and a U-shaped design, positioning it around the patient’s head. Made of homogeneous wax with a WET of 5.5 cm, the UB created an air gap of 2–8 cm, depending on the patient’s head size and position. The use of the UB demonstrated that reducing the air gap decreased lateral beam scattering, highlighting the importance of proximity between the bolus and the patient’s body.

An innovative approach to this problem involves the application of patient-specific proton beam compensators (BCs). This solution enables the complete reduction of the air gap between the compensator and the patient by tailoring the compensator’s shape to the patient’s body surface and customizing its thickness to match the target lesion’s location. 3D printing is the proposed technology for producing these personalized compensators. 3D printing is already in clinical use for the fabrication of patient-specific devices such as boluses, compensators, phantoms and immobilization items [13–24]. This technique is relatively inexpensive and simple.

The potential benefits of employing printed, individualized BCs to minimize the air gap in proton radiotherapy using PBS were demonstrated in [24]. Monte Carlo simulations supporting this approach showed a significant reduction in beam scattering when personalized compensators were used compared to conventional RS solutions. A case study [25] further underscored the utility of 3D-printed devices, showcasing the use of a printed helmet as a range discriminator, which effectively reduced doses to critical organs. While these studies highlight the potential of 3D-printed devices in proton therapy, our method goes further by tailoring the compensator design to the specific geometry and treatment requirements of each patient. This individualized approach ensures optimal dose conformity while accounting for the unique anatomical and treatment constraints of shallow tumors.

The use of 3D-printed materials in proton radiotherapy introduces unique challenges, particularly related to uncertainties in stopping power ratios (SPRs). Variability in material composition and density can lead to discrepancies between experimentally measured SPRs and values calculated by treatment planning systems (TPSs) using computed tomography (CT) calibration curves [14, 19, 26, 27]. To mitigate these discrepancies, it is necessary to override the Hounsfield Unit (HU) value for 3D-printed structures in the TPS. This process simplifies the material to a uniform value, disregarding internal heterogeneities, which may affect proton scattering and dosimetric accuracy. Quality control of filaments and printed accessories, and in particular their verification by performing CT, should ensure control of the impact of materials on the scattering of the proton beam.

The aim of this paper is to quantitatively assess the prospective benefits of the utilization of personalized 3D-printed compensators in the context of proton therapy for treating superficial tumors. We conducted a comparative in-silico analysis of treatment plans based on retrospectively selected data from six pediatric patients. This analysis was motivated by the observed clinical success of using 3D-printed compensators in three pediatric cases with superficial tumors, which were included in this study. The comparison focused on treatment plans that incorporated a RS placed at a certain distance from the patient’s body versus a BC placed directly on the patient’s mask. Alongside this plan comparison, the study also outlines the complete workflow for the application of a 3D-printed, patient-specific proton BC for PBS treatment targeting shallowly situated tumors. This comprehensive process includes material parameter assessment, ensuring printing uniformity, establishing the appropriate HU value for accurate matching, and designing and printing BC protocols, aimed at facilitating its clinical implementation.

## Materials and methods

### Patient data and planning objectives

Cyclotron Centre Bronowice (CCB) is a cyclotron-based proton facility with PBS technology (IBA Proteus-235 therapy system). The TPS Eclipse (version used 13.6) is provided by Varian Medical Systems [28].

This study was based on TPS simulations utilizing retrospectively selected CT data from six pediatric patients with diagnosed sarcomas located in the head and neck area, particularly in regions near the perioral area and eyes. Due to the superficial location of their tumors, these patients required the use of preabsorbing devices (RS or BC) to facilitate effective treatment. The gross tumor volumes (GTVs) for these cases ranged from 3.67 cm³ to 121.8 cm³, as delineated by physicians. The clinical target volumes (CTVs) were defined as the GTV plus a margin to account for microscopic disease spread. The planning target volumes (PTVs) were created by expanding the CTV with a 5 mm isotropic margin to address setup and proton range uncertainties.

A total relative biological effectiveness (RBE)-weighted (constant value 1.1) proton absorption dose of 50,4 Gy(RBE) and 55.8 Gy(RBE) was prescribed to the PTV. The prescribed fraction dose was 1.8 Gy(RBE) to be delivered in either 28 fractions or 31 fractions. Detailed information about patient characteristics and prescribed doses can be found in Table S1 in the Supplementary Data.

The following PTV constraints were applied: for the nominal plan, 98% of the PTV volume should receive at least 95% of the prescribed dose (D_98%_ >95%), and no more than 2% of the volume should receive more than 107% of the prescribed dose (D_2%_< 107%). The OAR constraints, presented in Table 1, depend on the radiosensitivity of the organs and are determined by the physician based on models and analyses in the literature [29, 30]. D_2%_ was used as an objective in the brainstem, spinal cord, chiasm, optic nerves, eyes and pituitary, the maximum dose D_max_ was used for the lens and tear ducts, and the mean dose D_Mean_ was used as an objective for the lacrimal glands and cochleas.

**Table 1:** Dose constraints for the organs at risks (OARs) used for the analyzed patients were verified and approved by the attending physician (abbreviations used: D_max,_ maximum dose; D_mean,_ mean dose; D_2%,_ dose to 2% volume of the organ)

Priority	OARs	Dose objectives
**1**	**Brainstem**	D_2%_ ≤ 54 Gy(RBE)
**2**	**SpinalCord/Canal**	D_2%_ ≤ 50 Gy(RBE)
**3**	**Chiasm**	D_2%_ ≤ 54 Gy(RBE)
**4**	**Optic nerves**	D_2%_ ≤ 54 Gy(RBE)
**5**	**Pituitary**	D_2%_ ≤ 45 Gy(RBE)
**6**	**Eyes**	D_2%_ ≤ 50 Gy(RBE)
**7**	**Lens**	D_max_ ≤ 6 Gy (RBE)
**8**	**Tear ducts**	D_max_ ≤ 10 Gy(RBE)
**9**	**Lacrimal glands**	D_mean_ ≤ 36 Gy(RBE)
**10**	**Cochleas**	D_mean_ ≤ 30 Gy(RBE)

### Configuration of the range discriminators

To degrade proton energy and shorten the path in the body, thereby obtaining proton energy suitable for the treatment of shallowly located tumors, two types of preabsorbers were used. The RS dedicated for this purpose, included in the IBA facility, is a uniform slab of Lexan (C_16_H_14_O_3_, ρ = 1.2 g cm^-3^) material with a thickness of 36.9 mm, which corresponds to 41.96 mm WET and consequently 1.14 water equivalent ratio (WER). The RS is permanently attached to the nozzle and can be placed or removed only from the beamline.

The second preabsorber used in this study was an individually designed, personalized BC placed directly on the mask to which the individual adheres due to the exact modeling of the compensator shape based on the patient CT images. Such an individual approach to preparing the BC enables precise adjustment of both the patient’s body and the dimensions of the irradiated lesion and allows us to avoid the air gap between the preabsorber and the patient. The BC was attached to the patient’s mask. The thickness of the BC was estimated at 4 cm though it could be adjusted to accommodate the depth and size of the tumor. Its weight varied around 1 kg, depending on the dimensions. The approach assumes a single compensator is utilized throughout the treatment cycle. In cases of minor anatomical changes, the treatment plan can be adjusted without replacing the compensator. Any plan modifications or decisions to produce a new compensator are evaluated by physicians and physicists to ensure effective treatment.

**Fig. 1:**
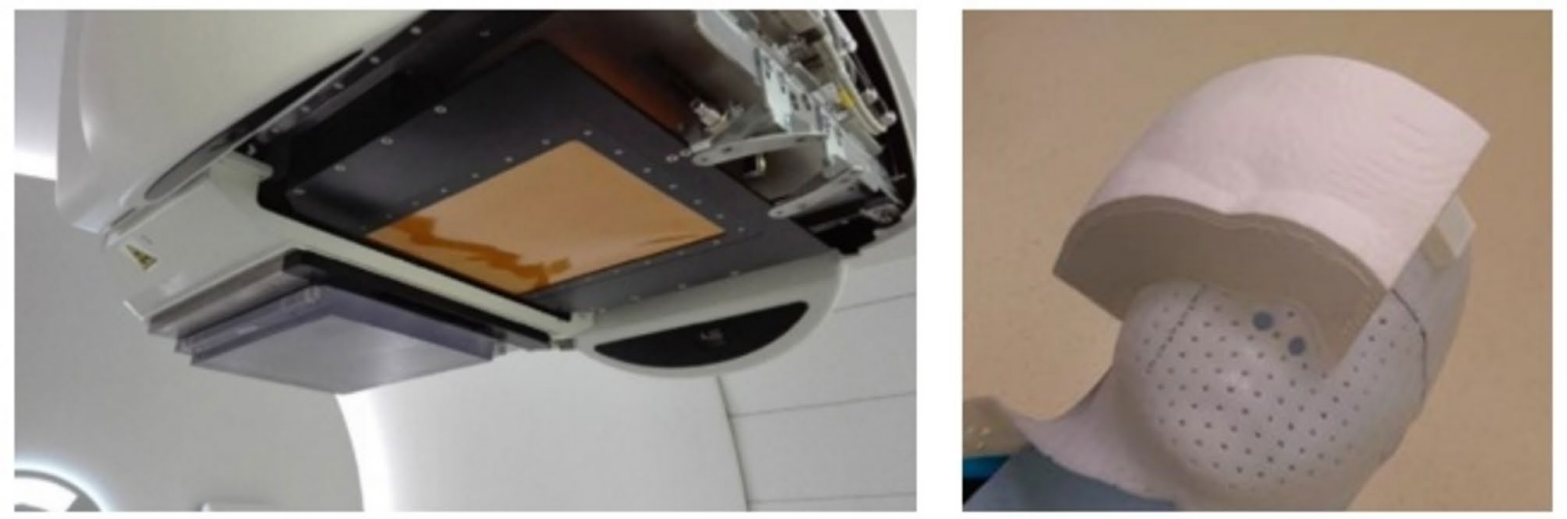
The configuration of the range discriminator used was as follows: the range shifter (RS) permanently attached to the beam nozzle (left) and the 3D-printed beam compensator (BC) attached to the patient’s mask (right)

Each configuration is shown in Fig. 1.

### Material properties and quality of the printed compensator

For the cases analyzed in this article, PLA thermoplastic material, which is a fully biodegradable aliphatic polyester produced by the Fiberlogy Company (Fiberlab S.A., Brzezie, Poland), was selected. The choice of this material was driven by its distinctive characteristics: (1) high density, which minimized the physical dimensions of the compensator; (2) ease of printing, which is especially crucial when using a 3D printer nozzle with a larger diameter (0.8–1.0 mm); (3) high availability; and (4) consistency of the material’s physical parameters. Its apparent density is 1.24 g/cm^3,^ and its chemical composition is similar to that of human tissue, with the molecular formula (C_3_H_4_O_2_)_n_. The material is insoluble in water and odorless, nonreactive and chemically stable if stored at room temperature and with limited exposure to light and moisture. All important data are collected in technical and material safety data sheets [31, 32].

The BC production process begins with the CT scan of the patient, taking into account all immobilization elements, especially the mask. The CT images are then exported to the TPS to design the shape of the compensator. Insets were added for permanent attachment to the patient’s mask. The final structure was exported in. stl format (a three-dimensional triangle mesh).

To ensure smoothness and eliminate surface irregularities resulting from CT-based numerical reconstruction, further adjustments were made to the compensator model. The adjusted file was exported in.gcode format, compatible with 3D printing software. Printing parameters, including bed temperature (35–45 °C), extruder temperature (230–240 °C) and cooling setting (need during whole printing process), were defined. Together with the compensator, the reference cube of the same material was printed. Standard radiotherapy markers were embedded in areas outside the treatment fields to enable precise positioning.

Compensators were printed for three of the six patients during the treatment stage and were used in irradiation after confirming their clinical applicability. For the remaining three patients, compensators were not printed during the treatment stage; however, these cases were selected for retrospective analysis to evaluate their potential use in treatment planning.

The compensators were printed via the fused filament fabrication (FFF) method with an ATMAT Signal XL printer. Printouts were made with a 0.8–1.0 mm 3D printer nozzle diameter and a resolution of 0.4 mm (layer height). he printing speed was set to 3700 mm·min⁻¹.

**Fig. 2:**
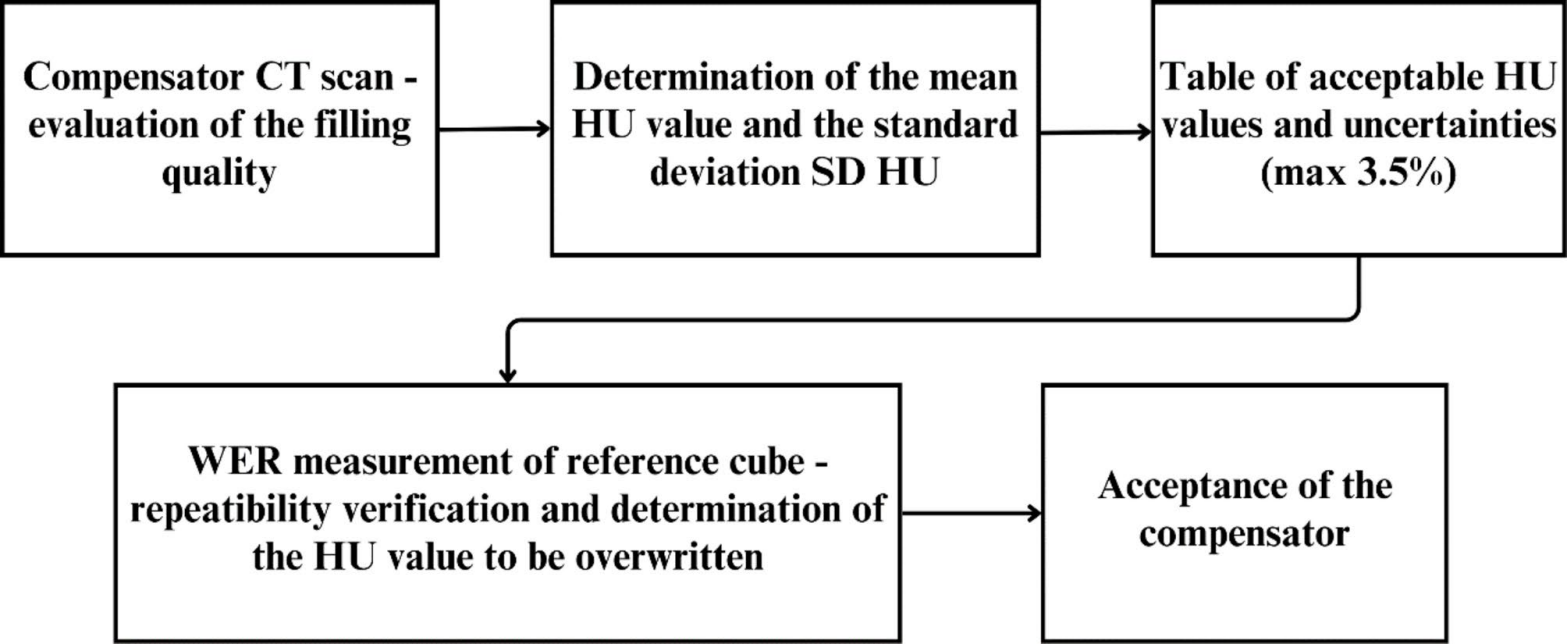
The scheme of the 3D-printed compensator acceptance procedure

The acceptance process for the compensator is illustrated in Fig. 2.

The first step involved a CT scan of the printed compensator to verify uniformity and detect potential holes or gaps. The average HU value and its SD were calculated to assess homogeneity. This step was critical because assigning an average HU value in the TPS results in the loss of heterogeneity details obtained from CT scans. Next, the HU values were compared with the stoichiometric calibration curve [33], permanently implemented in TPS and used clinically, to calculate the relative stopping power (RSP) uncertainty. The SD of the HU values should not lead to an RSP deviation exceeding 3.5% from the calibration curve. Table S2 (in Supplementary Data) provides the permissible HU deviation limits to meet this criterion.

For example, a compensator with a mean HU of 170 and an SD of ± 50 HU had a calculated RSP uncertainty of ± 2.16%, falling within acceptable limits. When all criteria were met, the compensator was approved for clinical use.

To validate the reproducibility of the printed material, reference cubes (4 × 4 × 3cm^3^) of the same PLA material as the compensators were prepared for WER measurements. The WER was measured by a Giraffe detector (as in subsection 2.3) to confirm material consistency. This approach allowed us to determine the actual value of the PLA material and facilitates the selection of the corresponding HU value, which was subsequently used to overwrite the BC volume in the TPS. The presented acceptance procedure was carried out on 3D-printed compensators for three of the six analyzed cases.

### Water equivalent range measurements

To ensure accurate dose calculations for treatment plans involving the beam compensator, comprehensive dosimetric measurements were conducted. The proton beam range was evaluated by measuring the WER of the beam after it traversed printed cubes made from the same polylactic acid (PLA) material used for the compensator. These measurements allowed to validate the consistency of the material’s stopping power. Beam characteristics were analyzed in the treatment planning system (TPS), including spot sizes, range uniformity, and penumbra width, to ensure accurate modeling and clinical applicability.

**Fig. 3:**
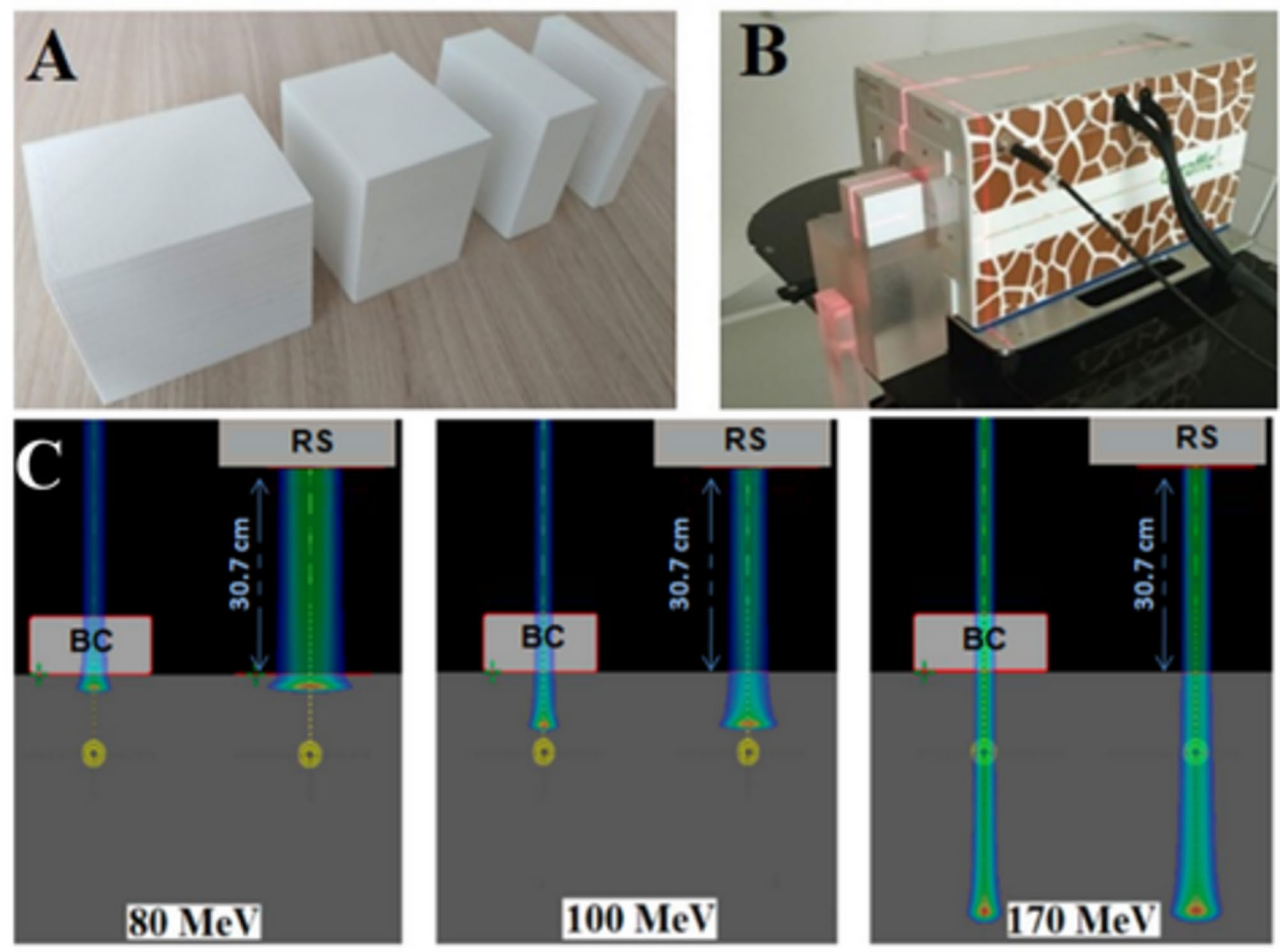
(**A**) Printouts prepared for Water Equivalent Ratio (WER) measurements. (**B**) Setup for WER measurements with Giraffe detector. (**C**) Setup geometry implemented in the treatment planning system (TPS) for determining the transverse dimensions of proton beams with energies (from left): 80, 100, and 170 MeV after passing through range discriminators - printed beam compensator (BC) and range shifter (RS)

To properly determine the WER for the printed compensator, four cubes with thicknesses of 1, 2, 4 and 6 cm were printed, each with consistent lateral dimensions of 5 cm × 5 cm (Fig. 3A).

WER measurements were conducted using the Giraffe detector (IBA-Dosimetry, Schwarzenbruck, Germany). The range of the pristine Bragg Peak (*R*_*pristine*_) for different energies (130 MeV, 150 MeV, 170 MeV and 200 MeV) was measured. Subsequently, the range was measured after passing through the printed cubes (*R*_*sample*_). To measure a sample thickness of 5 cm, a 6 cm cube was measured twice rotated by 90° between measurements. The actual thickness of the cubes was measured in the beam-parallel direction (L_sample_) using a caliper. WER values were calculated by comparing the pristine beam range to the range after passing through the investigated cube using the following formula:

1 $$\:WE{R}_{sample}=\frac{{R}_{pristine}-{R}_{sample}}{{L}_{sample}}$$

Results for cubes of different thicknesses were averaged for each energy and the measurement uncertainty was calculated as double standard deviation (SD) (at a 95% confidence level). The use of double SD is a widely accepted practice in statistical analyses, particularly under the assumption that errors are normally distributed.

The measurement setup is illustrated in Fig. 3B.

### Beam parameter analysis

To evaluate the influence of reducing the air gap on beam performance, the lateral spot sizes and penumbra characteristics were analyzed for the two therapeutic configurations (with RS and with BC). These parameters were examined in the context of improving dose delivery accuracy for the analyzed treatment scenarios.

For spot size measurements, a TPS-based setup was designed. Single spots of various energies (80, 100, 120, 150 and 170) passing through an RW3 phantom (Slab Phantom, PTW Freiburg, Germany) were analyzed with the use of two range discriminators — with RS and a printed PLA cube, both placed on the phantom surface. Results were compared with spot sizes for selected energies in the open field. As a spot size, the full width at half maximum (FWHM) was determined and recalculated to sigma in air to align with the Gaussian beam representation commonly used in treatment planning systems. Determining such a profile in the TPS is subject to uncertainty related to dose determination at a point in the TPS, which amounts to 2% for homogeneous plans [28].

The RS (36.94 mm thick, with a 41.96 mm WET and WER of 1.14), was situated 30.7 cm from the phantom surface and 36.85 cm from the isocenter. A PLA cube, 35.47 mm thick (matching the RS WET) was placed on the phantom surface, 6.15 cm from the isocenter. Lateral spot sizes expressed as sigma in air for each energy source were calculated in the TPS. Figure 3C illustrates the setup and simulations for three selected energies.

For measuring the lateral penumbra p20-80 of the proton beams (defined as the distance between the 20% and 80% isodose lines in the transverse direction), the setup and range discriminators placements were identical to those used for spot size measurements. A 5 × 5 × 5 cm^3^ superficial cube target was created and a treatment plan providing uniform coverage with a high dose to the entire target volume was prepared. Penumbras p20-80 were measured at depths ranging from 0 to 5 cm within the target volume. Obtaining numerical values required determining dose–intensity profiles perpendicular to the beam axis, which is subject to a 2% uncertainty [28].

### Treatment plan optimization and evaluation tools

After accepting the printed compensator, appropriate treatment plans were prepared utilizing two types of range discriminators. The BC concentration in the TPS was adjusted using an HU value derived from the dosimetrically determined WER, addressing the underestimation of SPR values from the calibration curve for the thermoplastic material.

The treatment plan configurations and proton beam parameters were chosen for each plan. The geometries of the compared plans with BC and RS could differ from one another, taking into consideration the recommendation to optimize the plans to the greatest extent possible. All plans employed 2 to 4 therapeutic fields, and no beam was allowed to directly traverse critical organs.

The target volume coverage for all PTV areas was evaluated as the first parameter for assessing the quality of treatment plans. For each patient, the volume of the PTV covered with the 95% isodose was determined. This analysis was performed to ensure that treatment plans met the established criteria for satisfactory coverage.

The plans were compared for each patient individually in terms of dose reduction in critical organs using the dose constraints specified in Table 1. Additionally, the dose distribution and uniformity of the target coverage were visually evaluated. For each plan, the conformity index (CI) for PTV was calculated using Eq. 2:

2 $$\:CI=\frac{{{TV}_{PIV}\left(98\%\right)}^{2}}{TV\:\bullet\:\:{V}_{RI}}$$

with TV as the target volume, TV_PIV (98%)_ as the target volume covered by the prescription dose (98% isodose) and V_RI_ as the total volume covered by the prescription dose. The CI parameter assesses how well the dose distribution conforms to the size and shape of the target volume [34]. A value closer to unity indicates a better match between the dose distribution and the target volume.

## Results

### Material WER determination

The WERs of the PLA material and the results averaged over energy and thickness are shown in Table 2.

**Table 2:** Water equivalent ratios (WERs) for the PLA material determined based on measurements with giraffe detector (expressed in mean value ± double SD) for different energies (E) and thicknesses (D)

		E [MeV]				mean +/- double SD
		200	170	150	130	
**D [mm]**	**60**	1.1827	1.1860	1.1844	1.1927	**1.1865 +/- 0.0087**
	**50**	1.182	1.186	1.1799	1.192	**1.185 +/- 0.011**
	**40**	1.188	1.188	1.1756	1.191	**1.186 +/- 0.013**
	**20**	1.194	1.184	1.1744	1.184	**1.184 +/- 0.016**
	**10**	1.188	1.169	1.1782	1.169	**1.176 +/- 0.019**
**mean +/- double SD**		**1.187 +/- 0.010**	**1.183 +/- 0.016**	**1.1785 +/- 0.0078**	**1.186 +/- 0.020**	**1.183 +/- 0.015**

Based on the conducted measurements, the average WER value of the PLA was 1.183. The uncertainty of this value was set as double SD (at a 95% confidence level), amounting to 0.015 (1.2%). The results, within the studied range, demonstrated the independence of the obtained WER value from the proton beam energy and material thickness.

### Beam parameter analysis

**Fig. 4:**
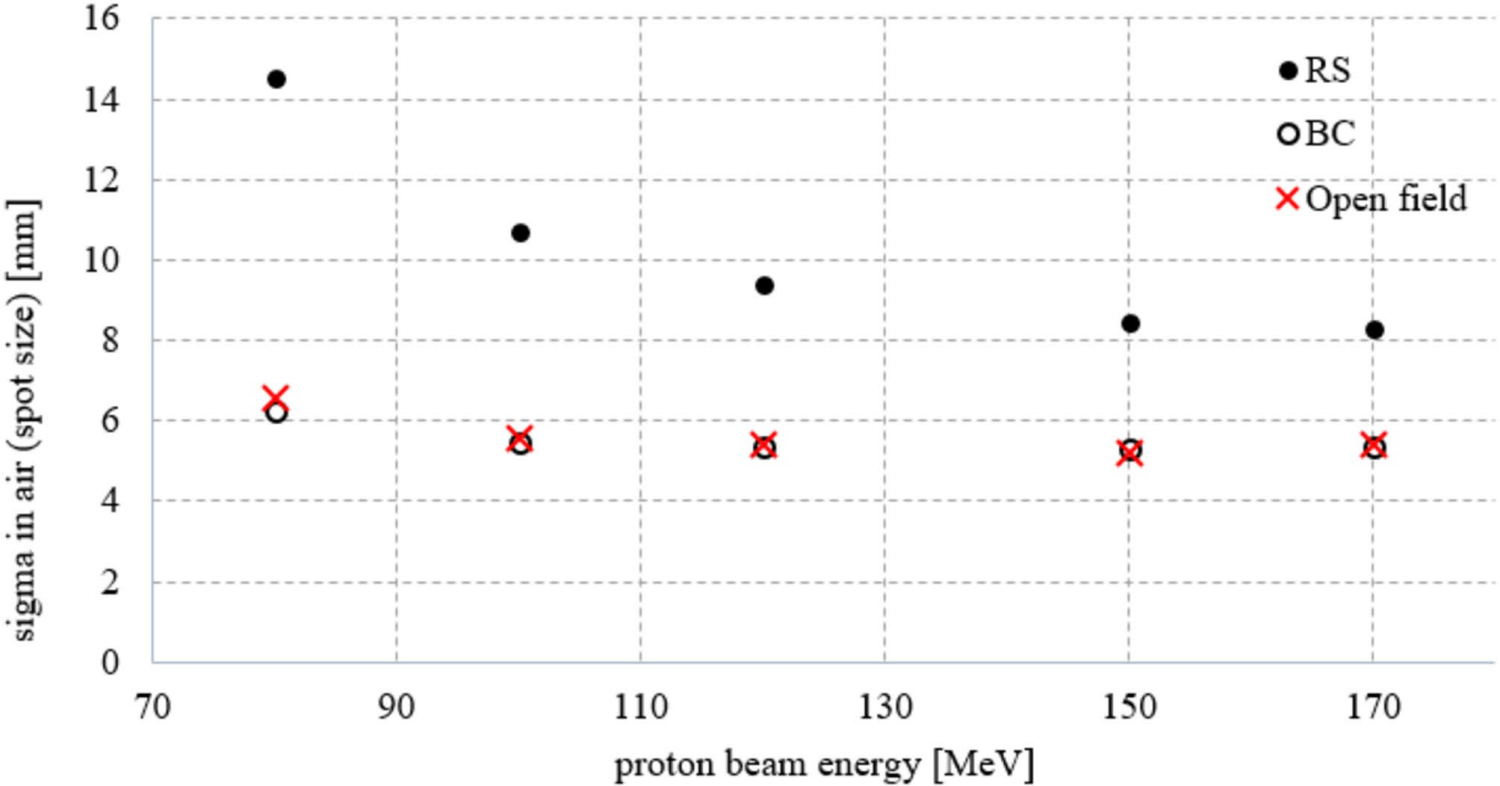
Spot sizes in terms of sigma in air determined in the treatment planning system (TPS) in the RW3 phantom for selected beam energies and two types of range discriminators– range shifter (RS) and 3D-printed proton beam compensator (BC). The results were also compared with spot sizes in the open field. The uncertainty in determining the spot size was 2% (for homogeneous plans) [28]

Figure 4 illustrates the results of lateral spot sizes obtained in the TPS for each proton energy. The graph shows a significant difference in the spot (RS: 3.419 mm, 2.517 mm, 1.957 mm, BC: 1.477 mm, 1.286 mm, 1.259 mm, for energies: 80 MeV, 100 MeV and 170 MeV, respectively). The use of BC allowed us to reduce the lateral sizes, especially for lower proton energies (57% reduction for 80 MeV, 49% reduction for 100 MeV, 36% reduction for 170 MeV).

**Fig. 5:**
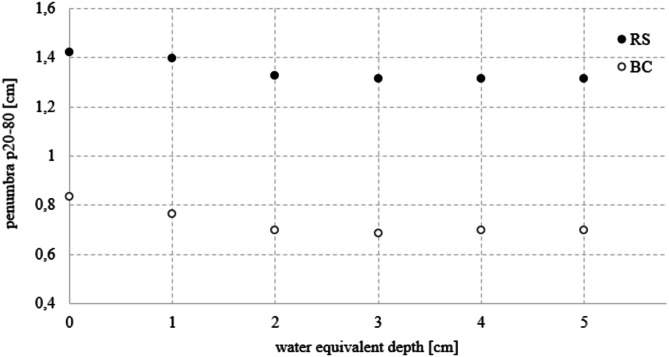
The penumbra p20-80 determined at different depths in target volume for two types of discriminators - range shifter (RS) and 3D-printed proton beam compensator (BC). The uncertainty in determining the penumbras is 2% (for homogeneous plans) [28]

Figure 5 shows the p20-80% penumbras determined at different depths within the target region (from 0 to 5 cm with a 1 cm increments). The graph illustrates the lateral penumbras demonstrating, that the use of BC resulted in narrower widths (for 1 cm depth: 45% reduction from 1.395 mm (RS) to 0.766 mm (BC), for 5 cm depth: 47% reduction from 1.314 mm (RS) to 0.698 mm (BC)). This narrowing corresponds to a steeper dose gradient in the transverse direction relative to the beam axis compared to the RS configuration.

### Physical properties of 3D-printed compensators

The three printed compensators had an RSP uncertainty below 3.5% (1.89%, 2.63% and 3.46% for case 2, 3 and 4, respectively), so they were accepted for use in treatment. After scanning the reference cubes and measuring the RSP (1.159%, 1.156% and 1.149% for case 2, 3 and 4, respectively), the HU value for each cube was determined (270 HU, 270 HU and 250 HU for case 2, 3 and 4, respectively) and subsequently used to overwrite CT numbers assigned to the compensator in the TPS. The standard procedure of converting HU to SPR, based on the implemented calibration curve in the TPS, cannot be directly applied for PLA material.

The detailed results of these evaluations are summarized in Table 3.

**Table 3:** Acceptance evaluation for each printed compensator - mean Hounsfield units (mean HU), standard deviation of HU (SD HU), relative stopping power (RSP) and RSP uncertainty

	3D printed beam compensator		
	Case 2	Case 3	Case 4
Estimated volume [cm^3^]	960	870	580
**mean HU**	156	144	137
**SD HU**	40	82	65
**RSP (based on calibration curve calculation)**	1.1038	1.1098	1.0945
**RSP uncertainty [%]**	1.89%	2.63%	3.46%
	**accepted**	**accepted**	**accepted**
measured RSP for reference cube (**± 1.2%)**	**1.159 ± 0.014**	**1.156 ± 0.014**	**1.149 ± 0.014**
CT numbers overwritten in TPS	**270**	**270**	**250**

### Plan quality comparisons

Across all analyzed cases and for both range discriminators (BC and RS), the coverage exceeded 99%, except for case 5, where the BC coverage was 97.7%. These results confirm that the plans met the criteria for satisfactory treatment planning.

For BC plans, the median coverage was 100%, with a range of 97.7–100% across all PTV structures. For RS plans, the median coverage was 99.6%, with a range of 99.1–100%.

**Fig. 6:**
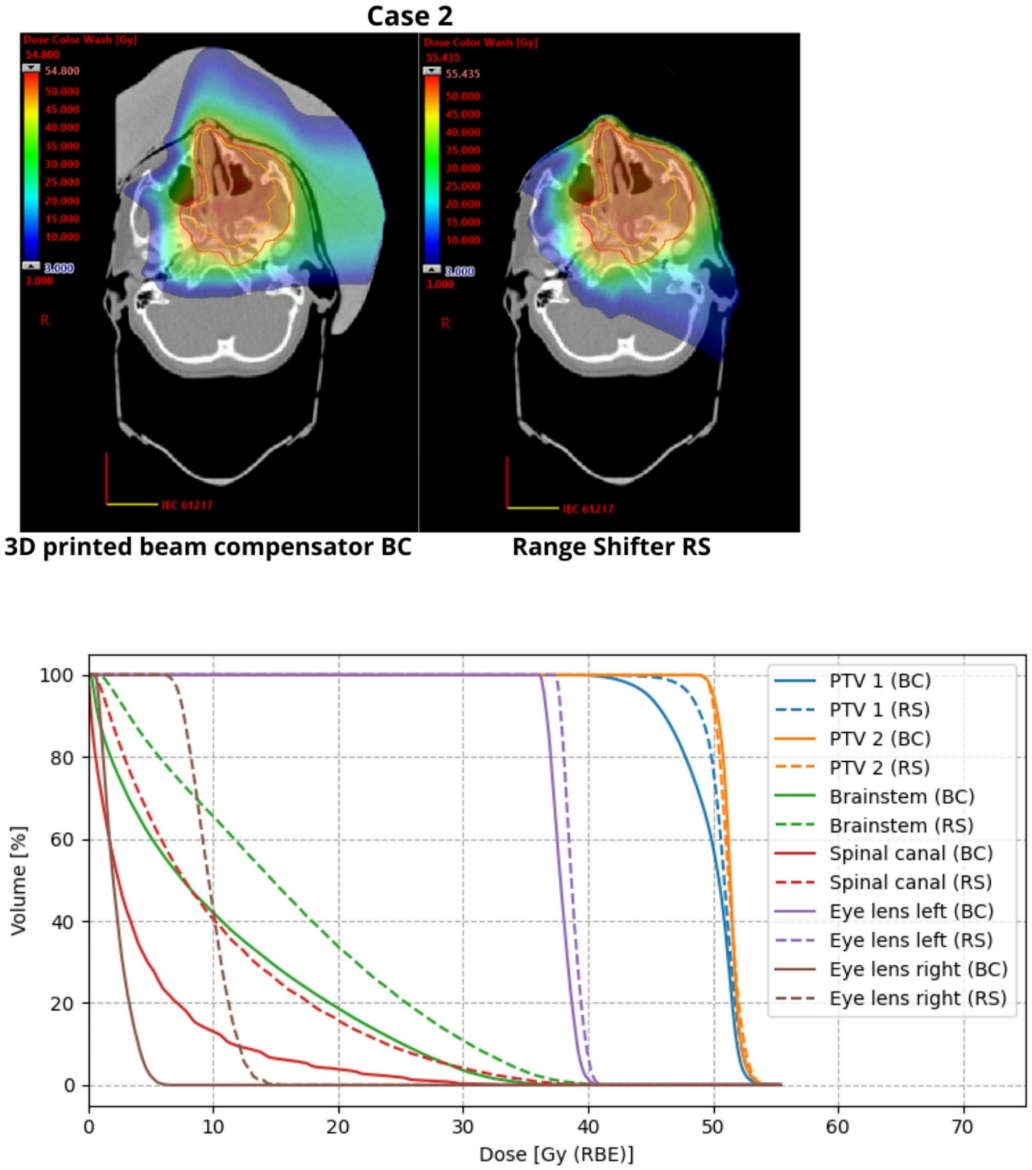
(Top) Dose distribution comparison example for case 2. The red contour represents PTV1, while the yellow contour represents PTV2. (Bottom) Dose-volume histograms (DVHs) for the target structures (PTV1 and PTV2) and selected organs at risk (OARs: brainstem, spinal canal, and eye lens) for case 2. The comparison between the 3D printed beam compensator (BC) and range shifter (RS) configurations

The dose distributions of all the modalities satisfied the acceptance criteria — no areas with excessively high doses (exceeding 110% of the prescribed level) were observed and critical organs received doses below established tolerances. For critical organs, the application of BC resulted in dose reductions, such as a decrease in the mean dose to the eye lens right by 8.9 Gy (RBE) (57.8%) compared to the RS modality. Figure 6 illustrates an exemplary dose distribution for one of the analyzed cases (case 2) and presents DVHs for the target structures (PTV1 and PTV2) and selected critical organs (brainstem, spinal canal and eye lens). The results for the remaining cases and selected OARs are provided in the Supplementary Data (dose distribution comparisons - Figures S1-S5, DVHs– Figures S6-S11).

Doses for OARs, calculated for all cases and plans using RS and BC, are presented in Table 4. For most organs and all analyzed cases, no deterioration in dose distribution was observed with the use of the BC. Instead, improvements were achieved, particularly in the form of dose reductions to critical organs (above 90% reduction for the right optic nerve (case 1), left eye lens (case 4 and 6) or right eye (case 5)**).** BC enabled reductions of several tens of percent for critical brain structures, such as the pituitary gland (65% in case 4), brainstem (59% in case 5), and spinal cord (52% in case 3). The results indicate that the BC contributes to a clinically meaningful reduction in OAR doses, but they should always be reviewed by oncologists to ensure their applicability.

**Table 4:** Doses for organs (in Gy (RBE)) at risk (OARs) calculated for all cases and treatment plans using range shifter (RS) and 3D-printed beam compensator (BC) according to dose constraints listed in Table 1. Bold font indicates cases where the use of the BC provided a benefit in comparison to the RS. The uncertainty in dose determination in the TPS is 3% for nonhomogeneous plans [28]

	OAR constraint	Case 1		Case 2		Case 3		Case 4		Case 5		Case 6	
OAR		BC	RS	BC	RS	BC	RS	BC	RS	BC	RS	BC	RS
Brainstem	D_2%_ ≤ 54 Gy(RBE)	-	-	**31.86**	**36.23**	**49.10**	**51.20**	34.89	34.22	**11.94**	**28.81**	-	-
Spinal Cord/Canal	D_2%_ ≤ 50 Gy(RBE)	-	-	**24.66**	**33.43**	**12.00**	**25.20**	**2.07**	**13.54**	**5.19**	**22.33**	-	-
Chiasm	D_2%_ ≤ 54 Gy(RBE)	0.10	0.10	**49.50**	**50.87**	**50.21**	**52.83**	**2.54**	**10.50**	**39.37**	**43.72**	2.534	0.66
Optic nerve L	D_2%_ ≤ 54 Gy(RBE)	**27.59**	**42.77**	-	-	**49.34**	**49.65**	**24.00**	**44.77**	-	-	**1.918**	**2.14**
Optic nerve R		**0.15**	**7.50**	-	-	**49.90**	**51.61**	**2.76**	**18.40**	-	-	49.50	48.64
Pituitary	D_2%_ ≤ 45 Gy(RBE)	-	-	**47.70**	**49.56**	**51.70**	**52.60**	**3.43**	**9.98**	**30.30**	**39.67**	**1.132**	**1.14**
Eye L	D_2%_ ≤ 50 Gy(RBE)	**49.70**	**52.00**	**49.73**	**49.96**	41.73	33.55	**19.75**	**38.82**	49.85	49.79	**0.315**	**2.06**
Eye R		**5.90**	**12.90**	**29.64**	**32.05**	**48.85**	**50.51**	**0.33**	**5.23**	**0.39**	**9.36**	**49.935**	**50.72**
Eye lens L	D_max_≤ 6 Gy(RBE)	-	-	**40.90**	**41.20**	**5.90**	**9.90**	**0.70**	**11.60**	**13.90**	**41.01**	**0.2**	**1.33**
Eye lens R		**1.70**	**6.40**	**6.50**	**15.40**	**13.80**	**42.60**	**0.10**	**0.80**	**0.10**	**2.90**	49.8	49.9
Tear duct R	D_max_≤ 10 Gy(RBE)	**9.90**	**27.80**	**44.60**	**48.60**	-	-	-	-	-	-	**15.4**	**38.05**
Lacrimal gland L	D_mean_≤ 36 Gy(RBE)	**27.20**	**39.20**	-	-	**0.16**	**1.22**	**0.14**	**8.65**	-	-	-	-
Lacrimal gland R		**0.05**	**0.78**	**0.57**	**1.29**	**9.82**	**18.14**	-	-	**0.004**	**0.26**	-	-
Cochlea L	D_mean_≤ 36 Gy(RBE)	0.05	0.05	**16.09**	**18.03**	**1.91**	**2.87**	-	-	**15.36**	**29.97**	-	-
Cochlea R		0.05	0.05	4.63	1.72	**16.21**	**22.29**	**0.10**	**0.45**	**0.10**	**0.45**	-	-

The conformity indexes, *CI*, (Eq. 2) calculated for each prepared treatment plan are presented in Table 5. For all the cases and analyzed PTV structures, the *CI* was closer to one for plans with BC as a preabsorber than for plans with RS.

**Table 5:** The conformity index (CI), calculated for each analyzed case for planning target volume (PTV) structures, for both beam compensator (BC) and range shifter (RS)

	Case 1		Case 2		Case 3		Case 4		Case 5		Case 6	
	BC	RS	BC	RS	BC	RS	BC	RS	BC	RS	BC	RS
**PTV1**	0.73	0.66	0.90	0.83	0.864	0.75	0.85	0.83	0.84	0.78	0.75	0.70
**PTV2**	0.63	0.62	0.83	0.61			0.70	0.65	0.80	0.58	0.70	0.66
**PTV3**	0.78	0.65										

## Discussion

This article outlines a comprehensive procedure for fabricating proton beam compensators through 3D printing technology, subsequently validated for pediatric cancer cases. A comparative analysis was conducted employing two types of range discriminators — RS and BC. The primary objective of using a customized compensator was to obtain the smallest possible spot size while ensuring proton energy compensation, which is critical for precise pediatric treatments.

Despite promising outcomes, the proposed approach has certain limitations, including potential delays caused by production time The total time required for model preparation, 3D printing, and quality testing is approximately one week, although additional delays may arise in cases of print failure requiring reprinting. The estimated cost of producing and printing a compensator in-house is relatively low, ranging from 50 to 100 USD, whereas outsourcing the process to an external company incurs higher costs, estimated at 200 to 300 USD. To enhance the method’s consistency and reliability for clinical application, future developments should focus on the utilization of certified medical-grade materials that comply with medical standards.

To accurately calculate the scattering of the proton beam after passing through this material and, consequently, determine the dose in the treated volume and individual critical organs, it was necessary to overwrite the material in the TPS with the mean HU value corresponding to the actual SPR parameter value determined through dosimetric measurements. Similar observations were also reported in the literature [14, 19, 26]. This method of introducing material parameters into the radiotherapy system underscores the importance of the compensator’s acceptance and quality assessment procedure. The print must fulfill requirements concerning homogeneity and print precision (describing in Sect. 2.3*The compensator acceptance procedure*), as after overwriting its volume with a predefined HU value, any heterogeneity information is lost.

The standard stoichiometric CT calibration curve correctly calculates the *SPR* for tissue-equivalent materials but is not suitable for nonwater-equivalent materials like those used for printed materials. Studies have suggested that dual-energy CT (DECT) could enhance the calibration procedure in proton therapy by providing more accurately *SPR* determinations for various materials [35–39]. This approach offers the potential to address the discrepancies associated with using standard calibration curves for printed materials. Research based on DECT calibration procedures [39], such as those proposed by Saito and Sagara [38], has demonstrated improved conversion of HU to SPR for nonstandard materials (compliance with measurements below 1%). These findings highlight the value of incorporating advanced imaging techniques like DECT to refine the estimation of SPR values for 3D-printed components, ensuring greater consistency and accuracy in their application in proton therapy.

High print quality is an essential prerequisite when using printed components for radiotherapy. The better the homogeneity of the print, the lower the dispersion of the proton beam obtained. First and foremost, an appropriate set of parameters for the available 3D printer must be selected to ensure the densest possible filling. The process of printing such large components is time-consuming, making printer stability equally significant. Moreover, FFF print analysis has indicated that prints can contain geometric defects and void spaces with volumes of up to 13% [40].

One potential approach to enhancing print homogeneity could involve printing only the contour and subsequently filling it with a substance similar in parameters to water. This approach results in greater homogeneity, albeit constituting a component composed of two materials. In this case, the contour must be sufficiently thick to ensure integrity, and its presence cannot be disregarded. This expands the possibility of adapting compensator parameters to radiotherapy requirements and enables the use of materials better simulating human tissues than thermoplastics. Such an approach was applied in the study by Canters et al. [15]. for irradiating nonmalignant skin tumors via electron beam radiotherapy; the authors printed the bolus outline and filled it with silicone rubber. Similarly, Ehler et al. [41]. printed a phantom outline for their research, filling it with a homogeneous mixture of tissue-like material. A similar solution, i.e., filling the thyroid phantom outline with water to improve homogeneity, was considered in the discussion of Alssabbagh et al. [42].

In proton therapy, in addition to the dose distribution around the treated lesion, understanding the doses affecting more distant organs is of paramount importance. Such doses stem from scattered radiation, predominantly consisting of photons and neutrons but also from dispersed charged particles [43]. Scattered radiation analysis is an important aspect of radiotherapy, as it can induce secondary malignancies in tissues and organs beyond the irradiated area, especially in children [44, 45]. In proton radiotherapy facilities utilizing the PBS system, the majority of secondary radiation arises from interactions between protons and the patient’s body. However, beam modifiers can also constitute a significant source of secondary radiation [46].

The use of a printed compensator raises the question as to whether the resulting scattered radiation surpasses that encountered when using range shifters. This matter was thoroughly examined in the work of Wochnik et al. [46]., who ultimately concluded that the doses received by critical organs are either equivalent or lower when utilizing printed bolus compensators as opposed to RS. Moreover, the measured neutron ambient dose equivalent at a distance of 2.25 m from the isocenter was on the order of several tens of µSv for doses of 50 Gy throughout the treatment cycle. This is a very low value, comparable to that of exposure on a single transatlantic passenger flight. Similar doses are reported in Mares et al. [47]., who led the authors to the thesis that in justified cases, parents could safely stay in the therapy room while their children are irradiated.

The literature contains various approaches to minimize airgaps in the treatment of shallow lesions. Some centers employ specialized equipment, such as movable range shifter [48, 49], while others propose more universal approaches like universal U-shaped boluses [12, 49]. Although these methods do not completely eliminate the air gap, they significantly reduce spot sizes and offer versatility for different clinical scenarios.

Studies highlight the impact of these approaches on beam characteristics. For instance, the universal U-shaped compensator described by Both et al. [12]. achieved spot sizes comparable to those in an open field for a 110 MeV proton beam (e.g., 5.5 mm and 5.9 mm vs. 5.3 mm in the open field). By contrast, RS-IBA systems resulted in larger spot sizes, such as 14.9 mm under similar conditions. Movable RSs [48, 49] reduce air gap to approximately 15–20 cm, which substantially decreases beam scattering and spot sizes (e.g., for 80 MeV, spot sizes were reduced to 9 mm and 12 mm for air gaps of 17.5 cm and 30.5 cm, respectively).

The BC approach described in this study offers a distinct advantage by reducing the air gap to zero, resulting in a spot size of 6.4 mm for an 80 MeV proton beam. This capability enables beam characteristics that are closer to those of an open field, a result not achievable with movable RS systems.

To improve dose conformity and penumbra sharpness in proton therapy, Winterhalter et al. (2018) proposed lateral fall-off optimization techniques [50]. These approaches focus on optimizing spot placement to enhance the sharpness of the dose distribution. Combining the use of BCs with lateral fall-off optimization could hold great potential for further improving dose distribution and minimizing risks to critical organs. However, integrating these techniques into clinical practice would involve addressing several challenges, such as increased system complexity and reduced flexibility in adapting to anatomical changes during treatment. These practical considerations must be carefully examined in future research to fully harness the benefits of combining both strategies.

## Conclusions

This study highlights the implementation of customized, 3D-printed proton beam compensators for proton pencil beam scanning for treating shallow pediatric tumors. Unlike conventional range shifter, BC was employed as a preabsorber in six clinical cases.

The results confirmed that the use of BC improved target coverage, dose distribution and dose sparing for organs at risk without any observed adverse effects during treatment. These results suggest that 3D-printed proton beam compensators are not only feasible but also provide significant benefits in treatment planning for shallow lesions, especially in pediatric patients.

## Electronic supplementary material

Below is the link to the electronic supplementary material.

Supplementary Material 1

Supplementary Material 2

Supplementary Material 3

## Data Availability

All the data generated or analyzed during this study are included in this published article [and its supplementary information files]. Computed tomography images analyzed during the current study are available from the corresponding author upon reasonable request.

## Abbreviations

BC: Proton beam compensator
CCB IFJ PAN: Cyclotron Centre Bronowice at the Institute of Nuclear Physics
CI: Conformity index
CT: Computed tomography
CTV: Clinical Target Volume
DECT: Dual-energy computed tomography
DVH: Dose-volume histogram
FFF: Fused filament fabrication
FWHM: Full width at half maximum
HU: Hounsfield units
OAR: Organ at Risk
PLA: Polylactic acid
PT: Proton therapy
PTV: Planning target volume
RBE: Relative Biological Effectiveness
PBS: Pencil beam scanning
RS: Range shifter
RSP: Relative stopping power
SD: Standard deviation
SPR: Stopping power ratio
TPS: Treatment Planning System
UB: Universal bolus
WET: Water-equivalent thickness
