# Supplementary material for: Application of 3D-printed compensators for proton pencil beam scanning of shallowly localized pediatric tumors
Source: Radiat Oncol. 2025 Apr 29;20:66. doi: 10.1186/s13014-025-02646-3 (PMC12042327; doi:10.1186/s13014-025-02646-3)

**Application of 3D-printed compensators for proton pencil beam scanning of shallowly localized pediatric tumors**

**Agnieszka Wochnik^(1)^, Tomasz Kajdrowicz^(1)^, Gabriela Foltyńska^(1)^, Dawid Krzempek^(1)^, Katarzyna Krzempek^(1)^, Krzysztof Małecki^(2)^, Marzena Rydygier^(1)^, Jan Swakoń^(1)^, Paweł Olko^(1)^and Renata Kopeć^(1)^**

^1^Institute of Nuclear Physics Polish Academy of Sciences, Krakow, Poland

^2^University Children’s Hospital of Krakow, Poland

Corresponding author: [Agnieszka.Wochnik@ifj.edu.pl](mailto:Agnieszka.Wochnik@ifj.edu.pl)

1. Dose distribution comparison

Case 1


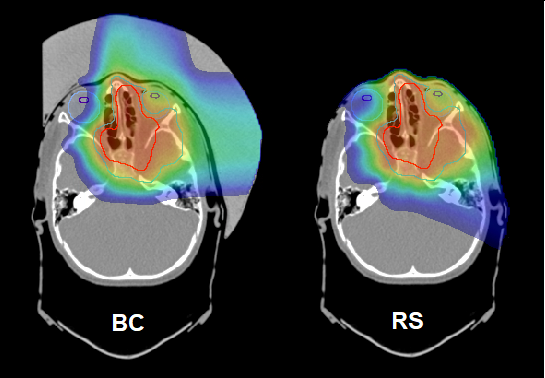


Case 2


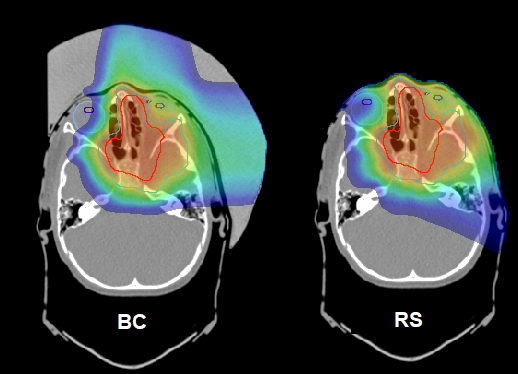


Case 3


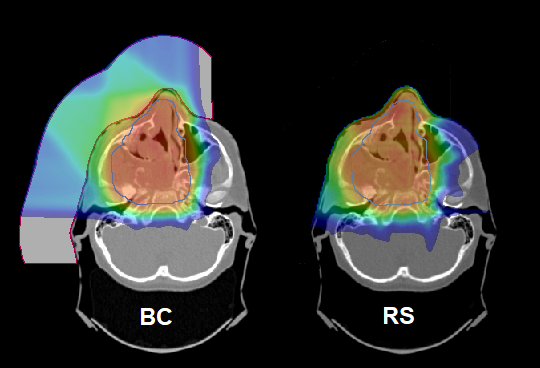


Case 4


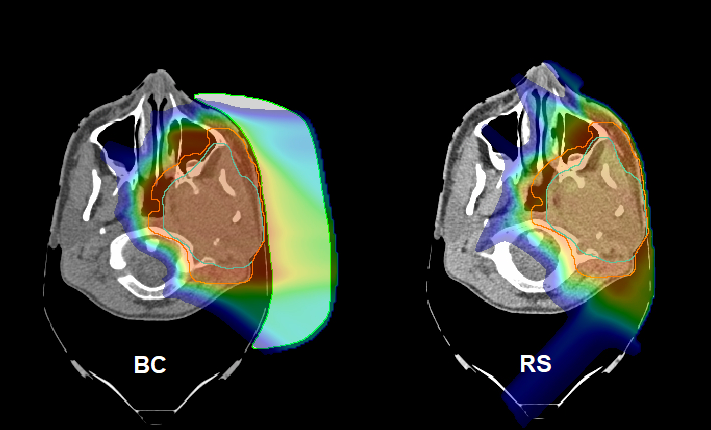


Case 5


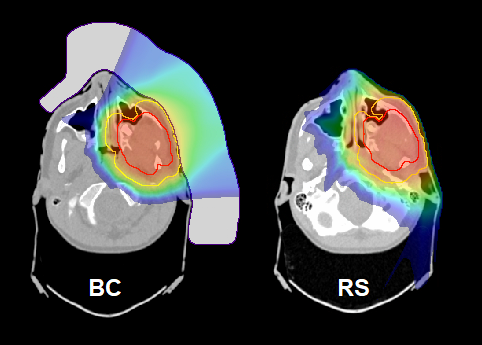


Case 6


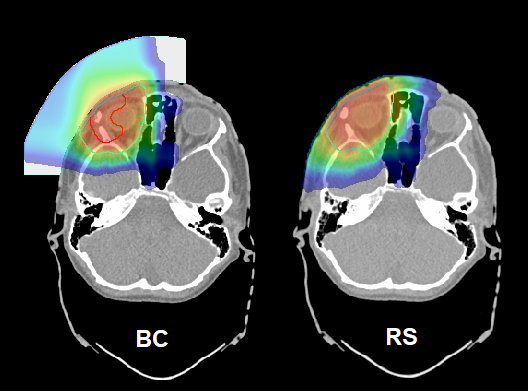


1. Dose-volume histograms

Case 1


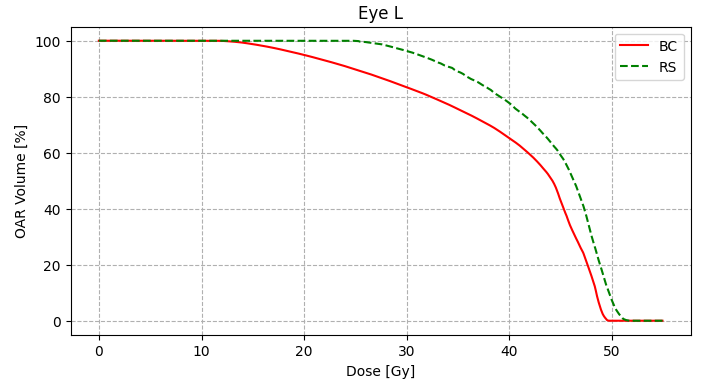


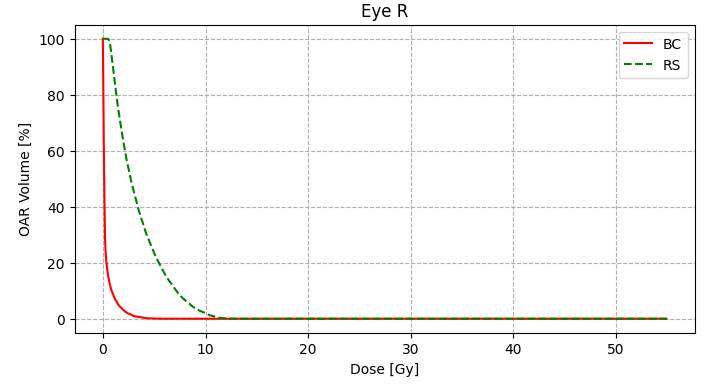


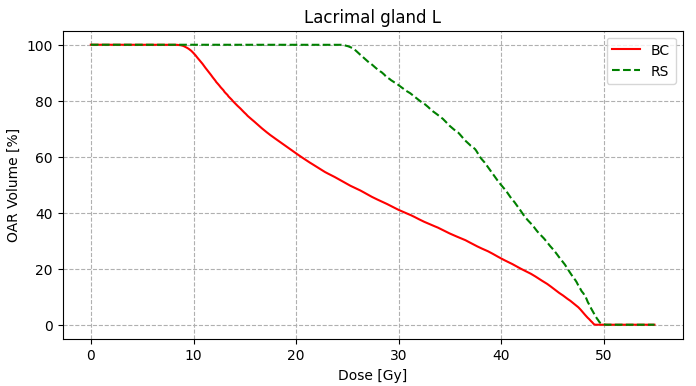


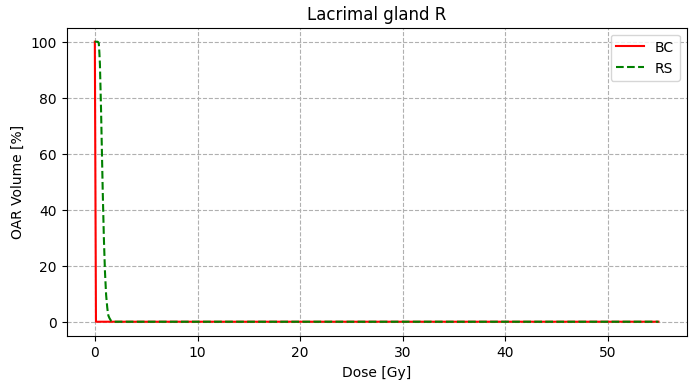


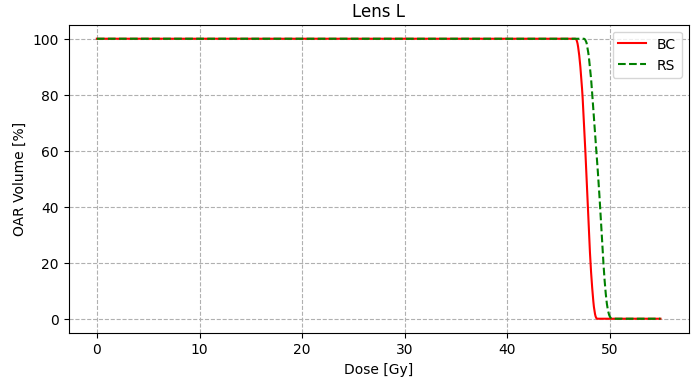


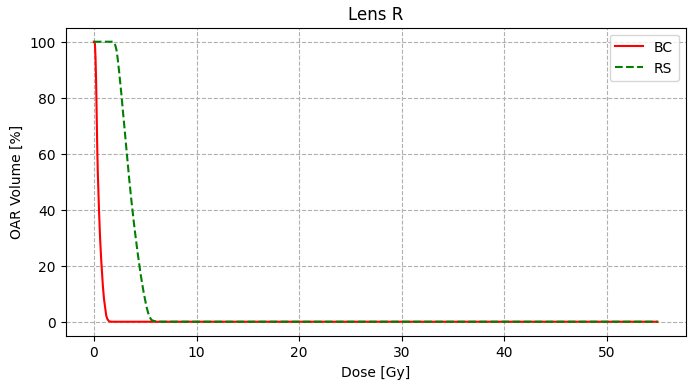


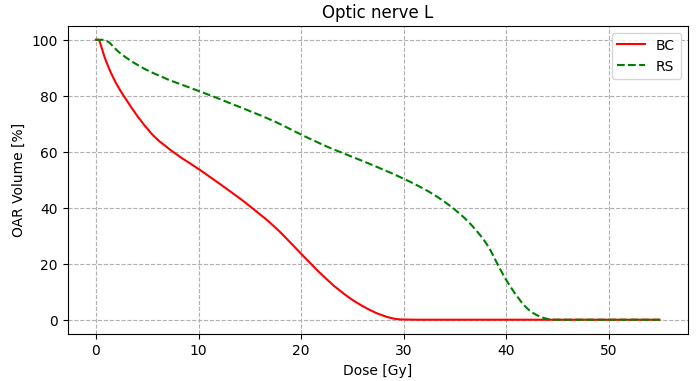


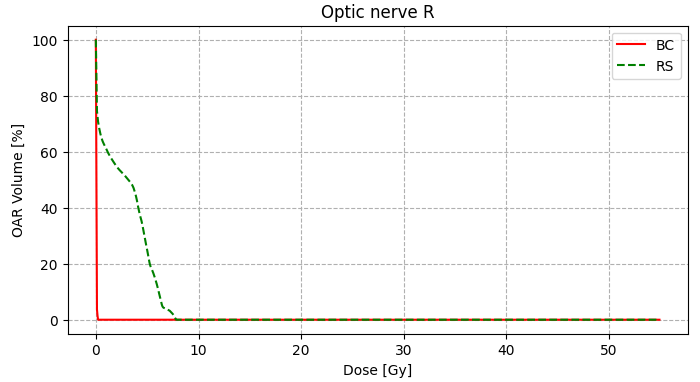


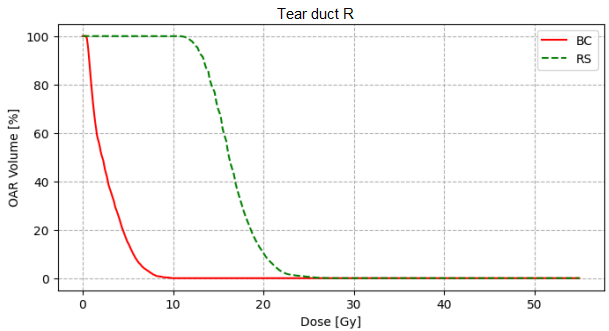


Case 2


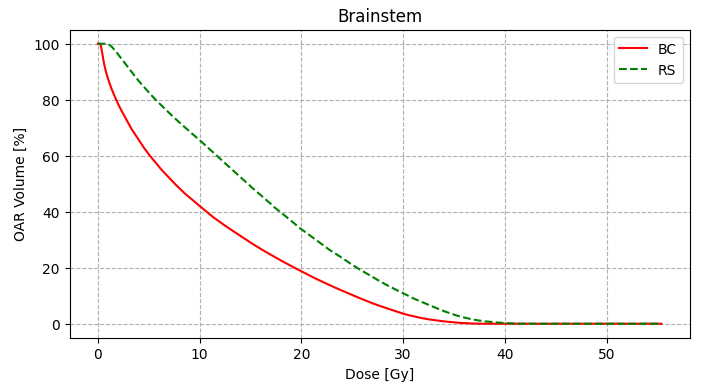


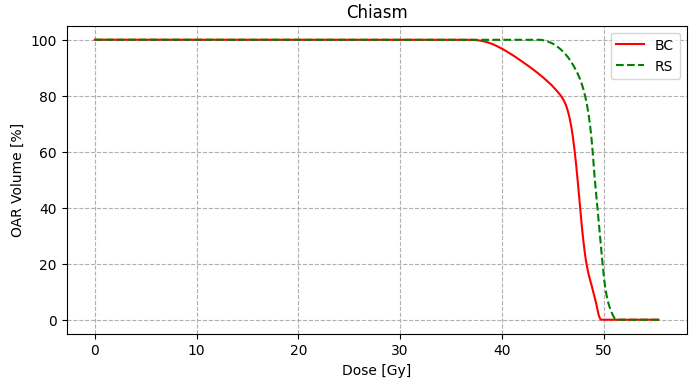


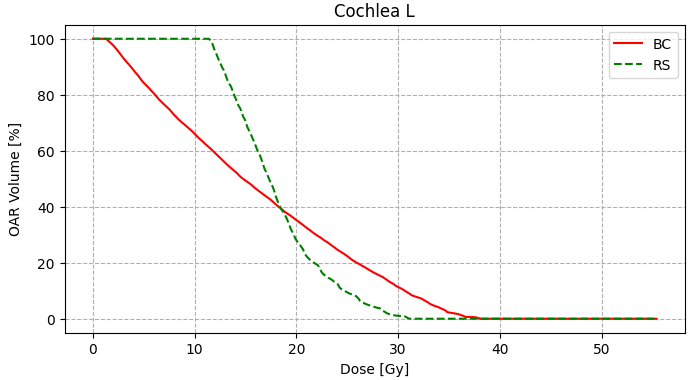


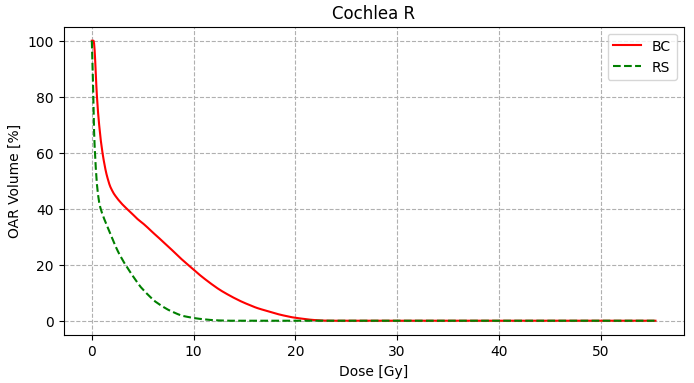


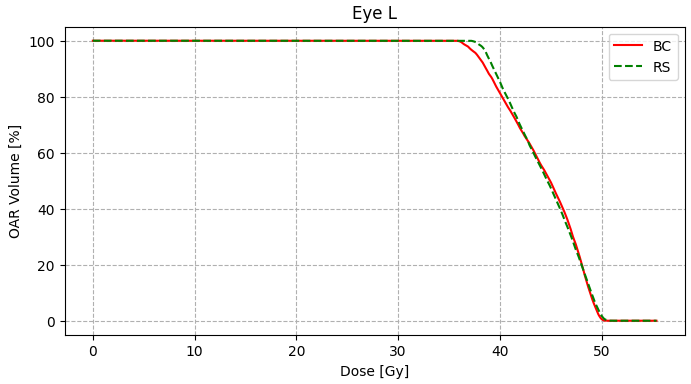


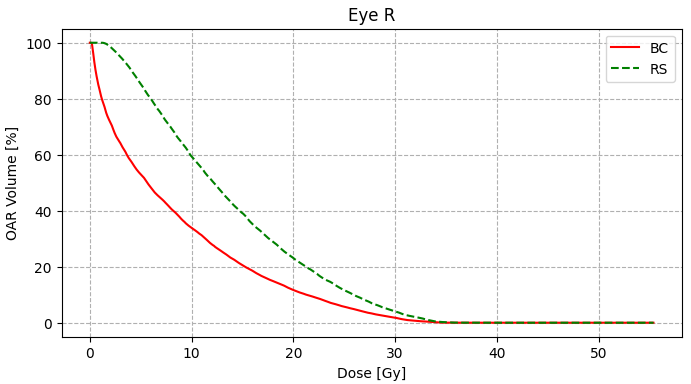


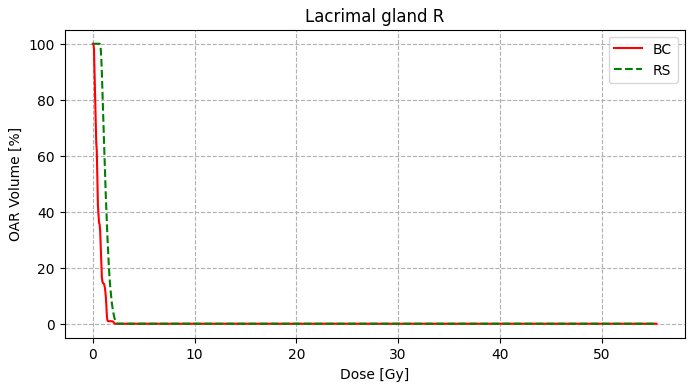


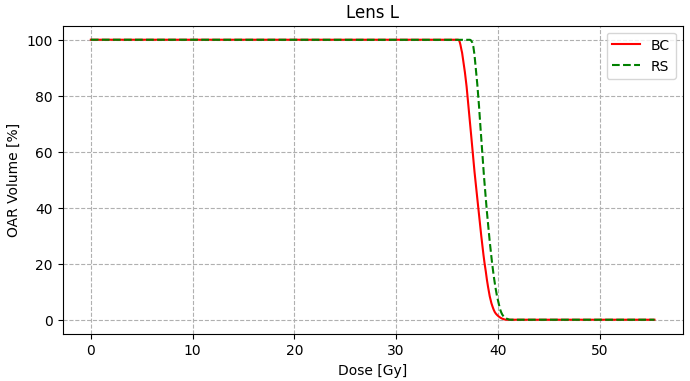


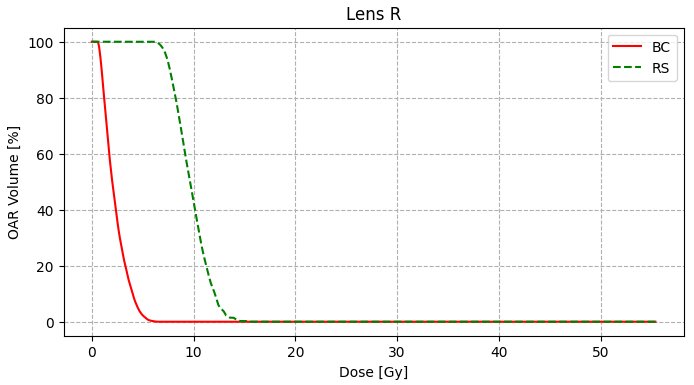


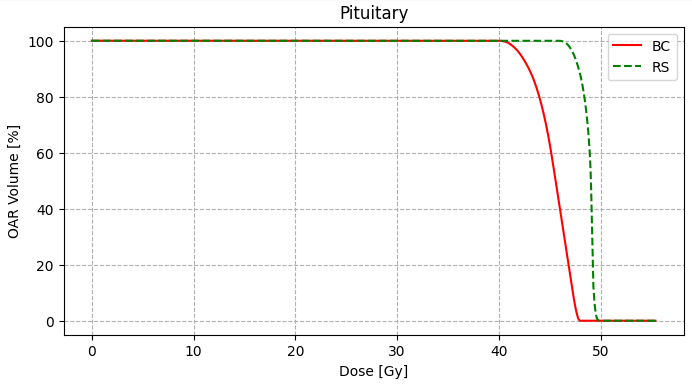


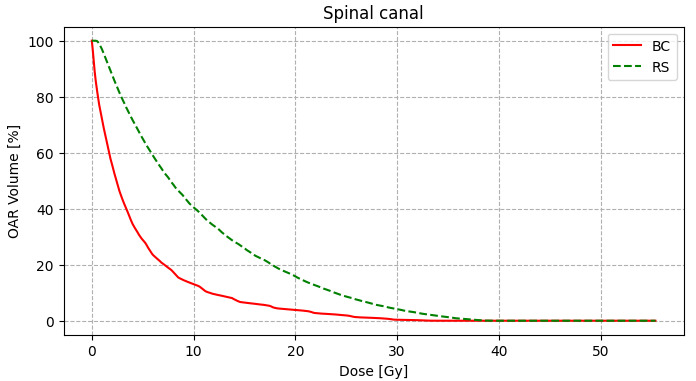


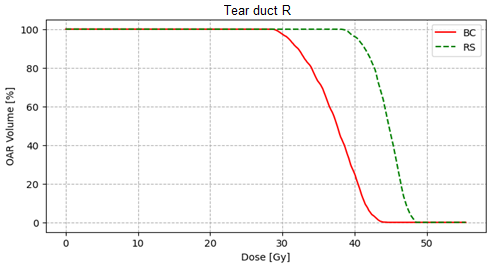


Case 3


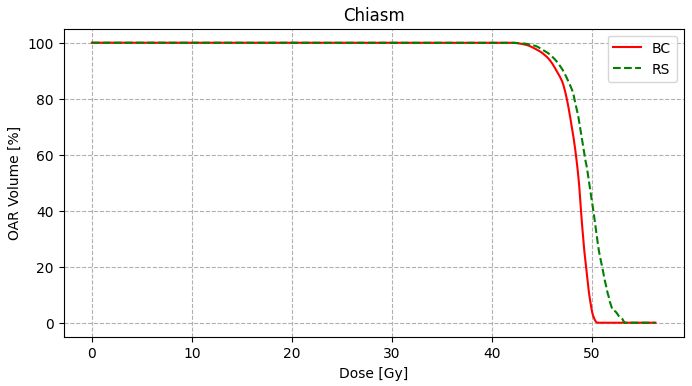

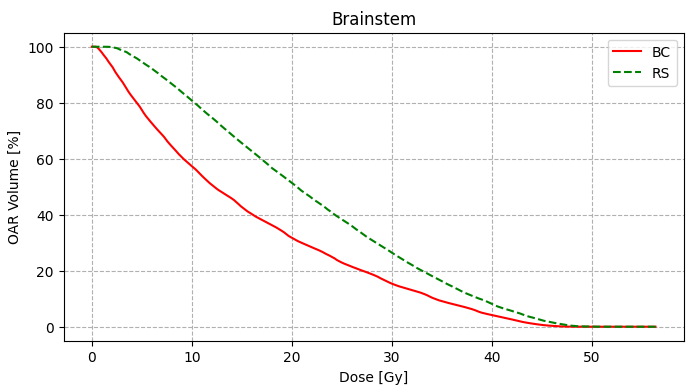


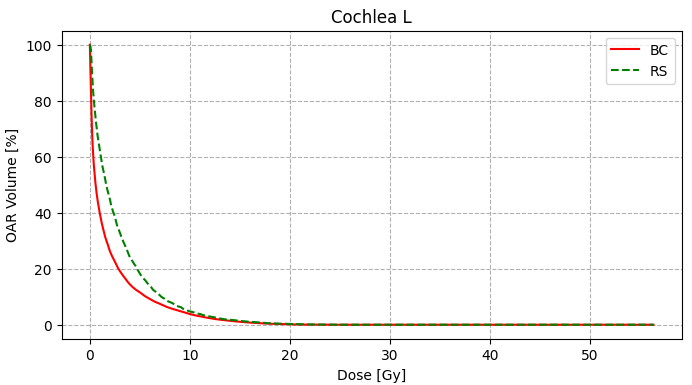


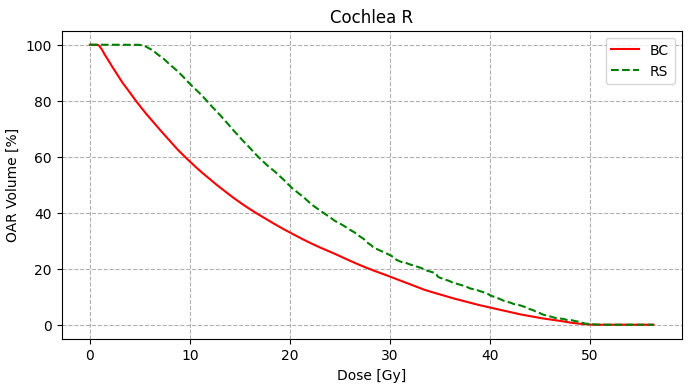


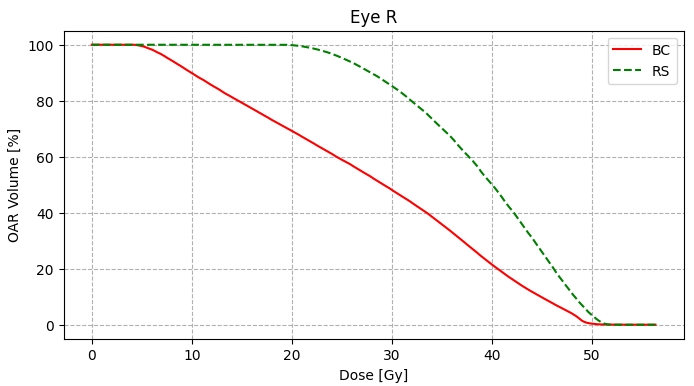


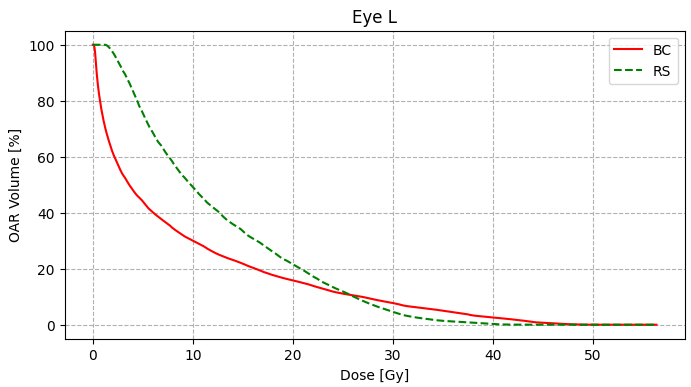


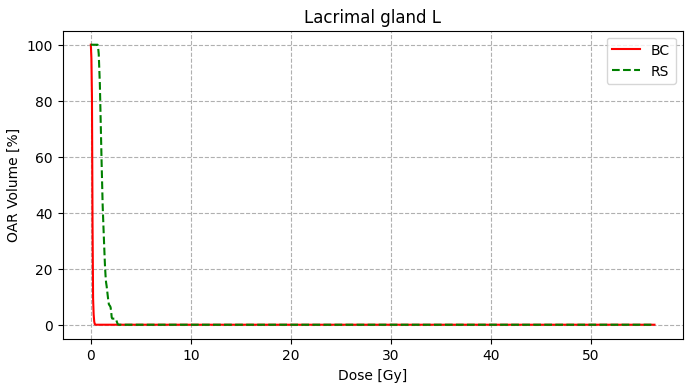


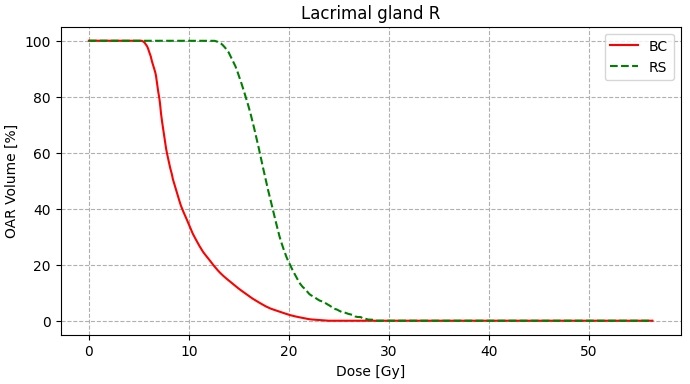


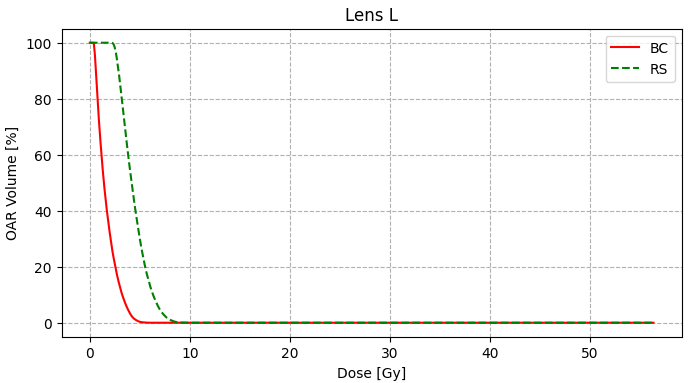


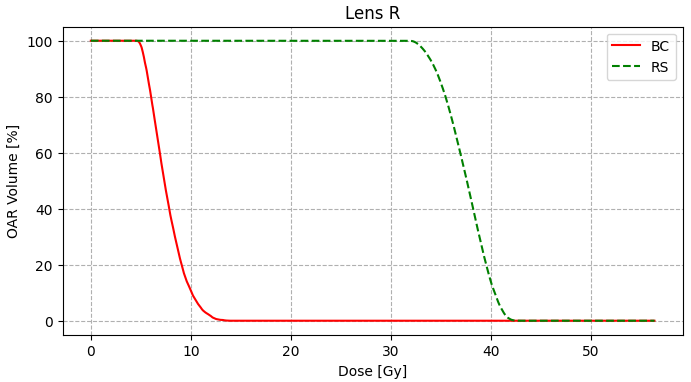


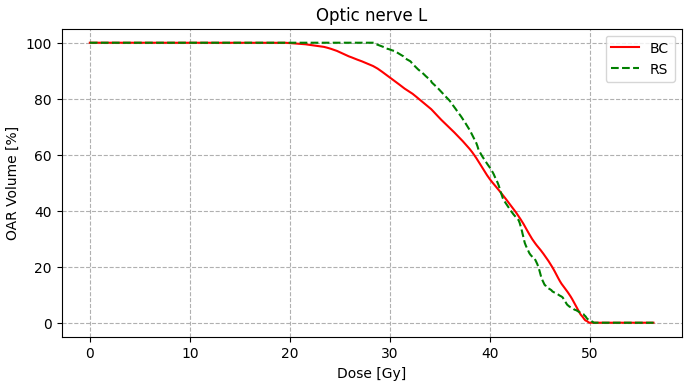


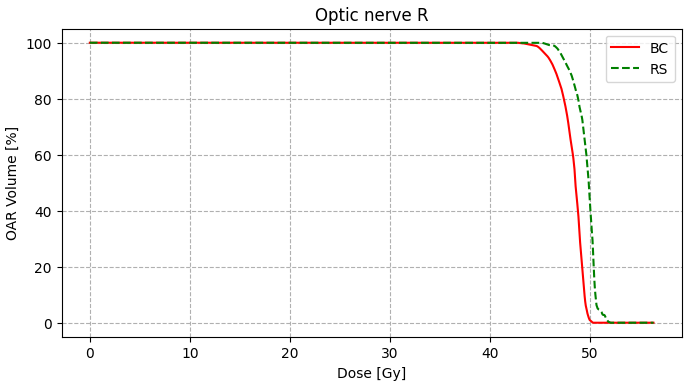


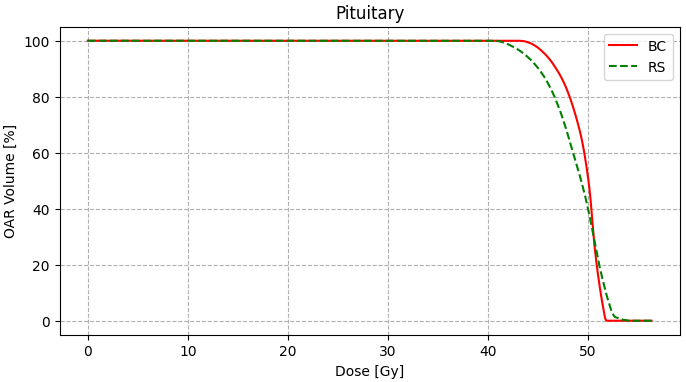


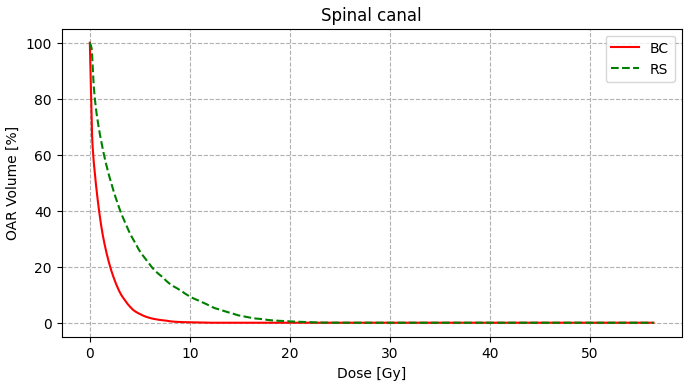


Case 4


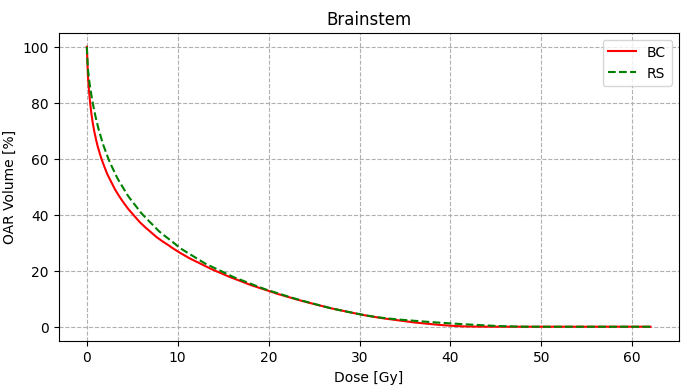


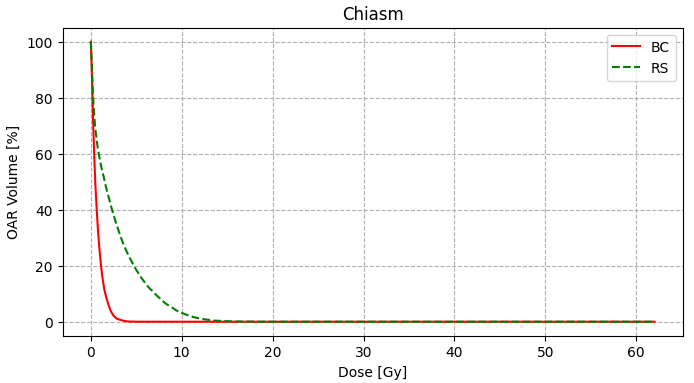


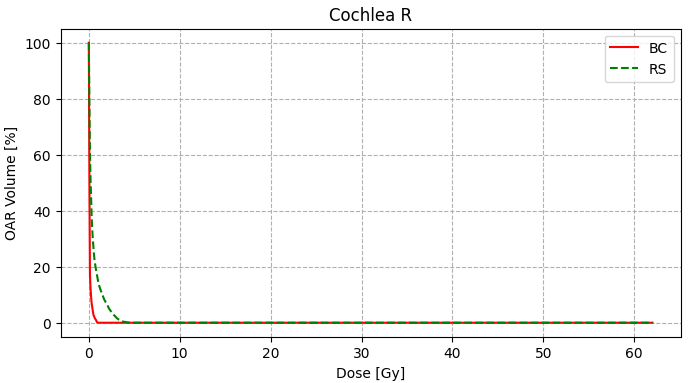


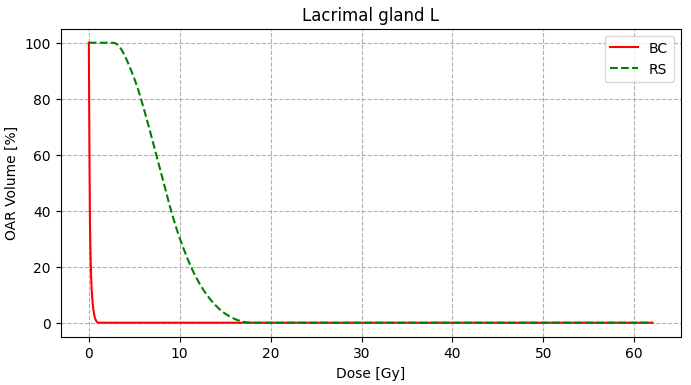


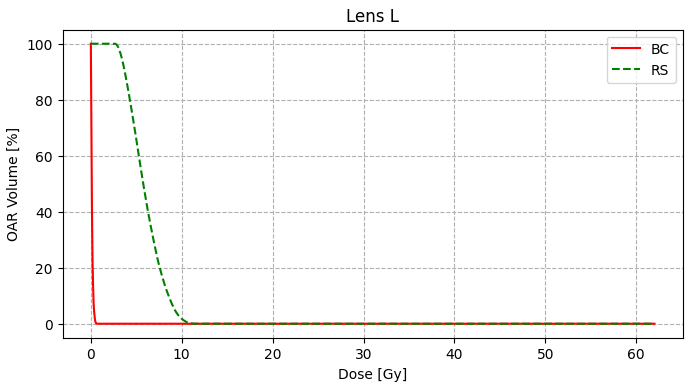


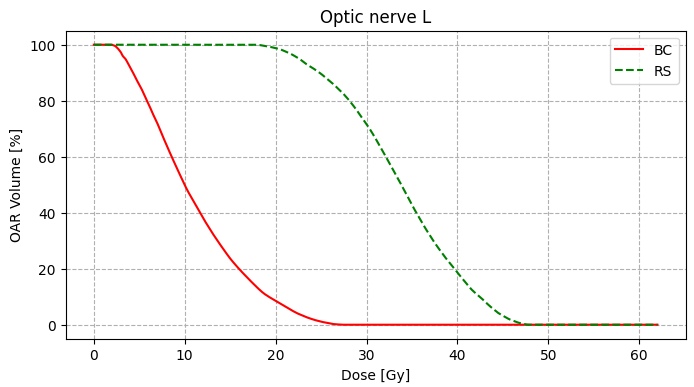


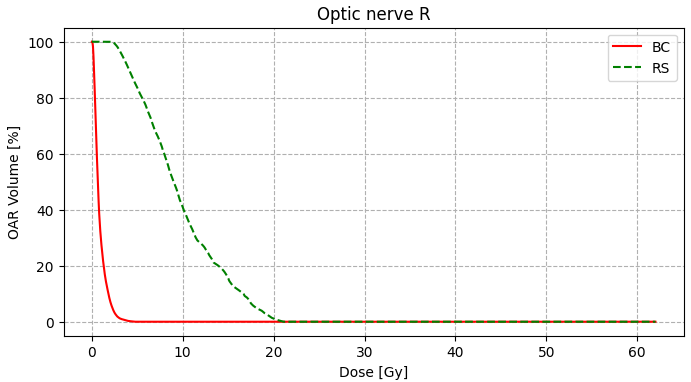


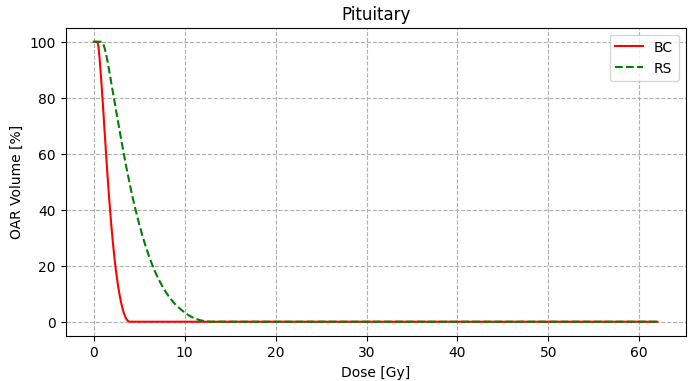


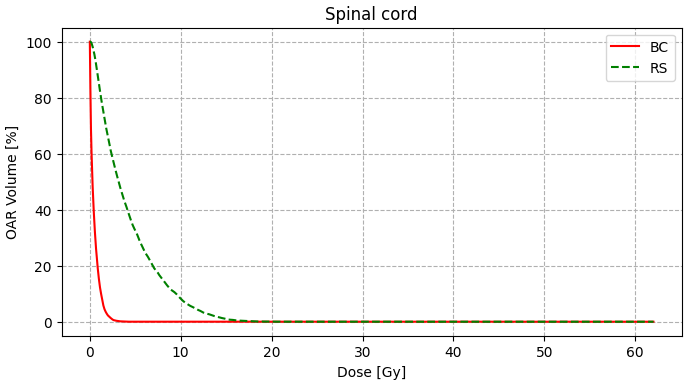


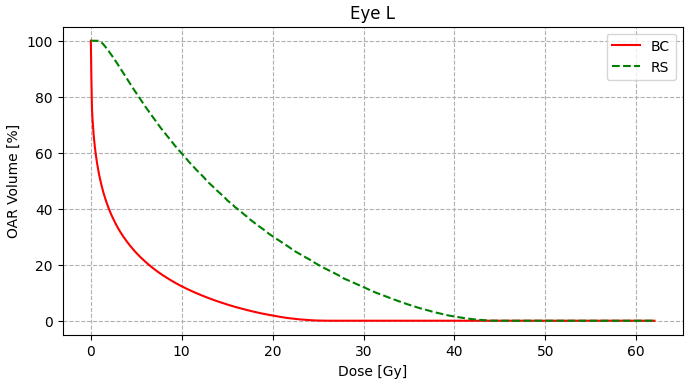


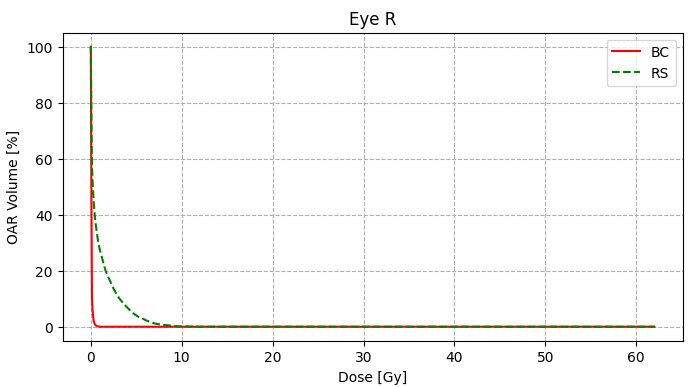


Case 5


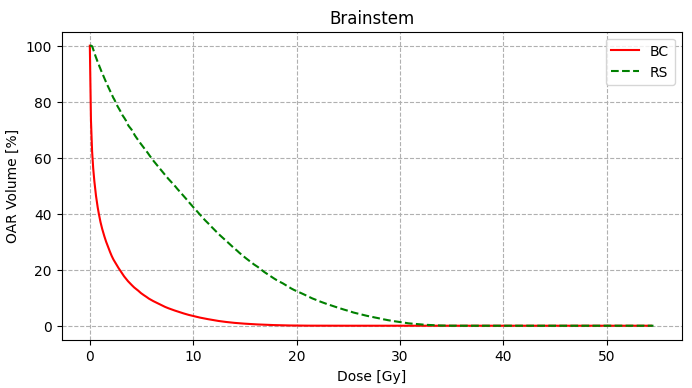


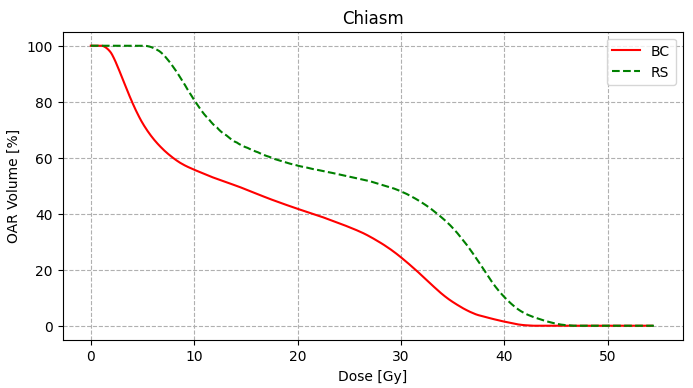


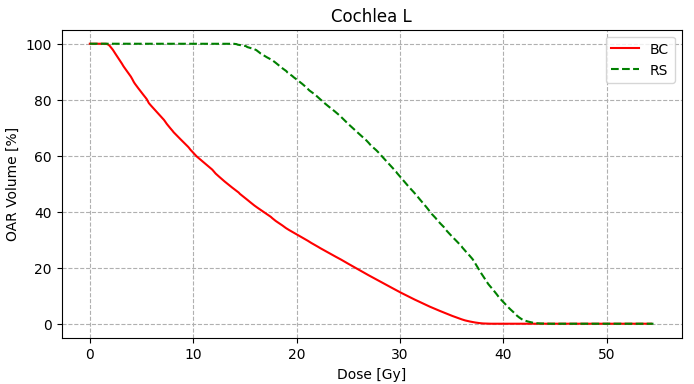


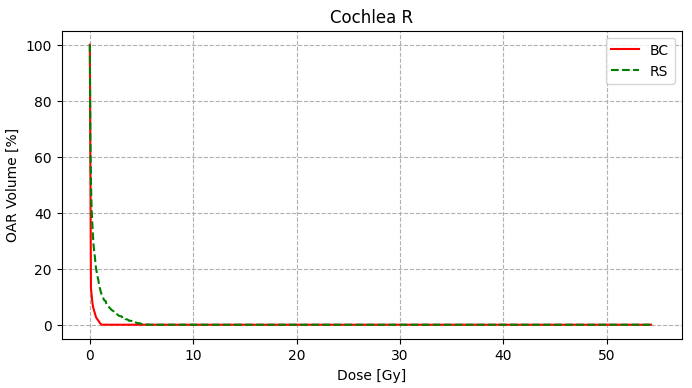


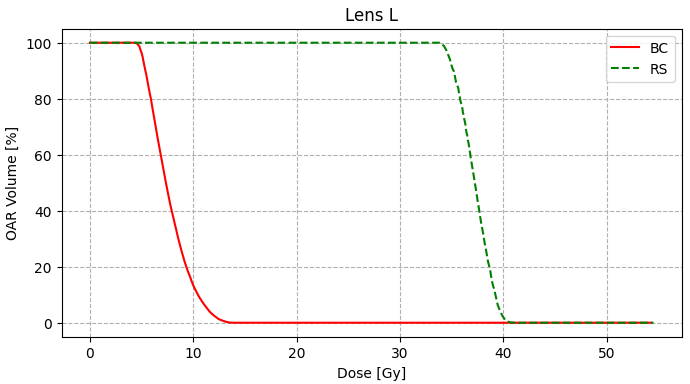


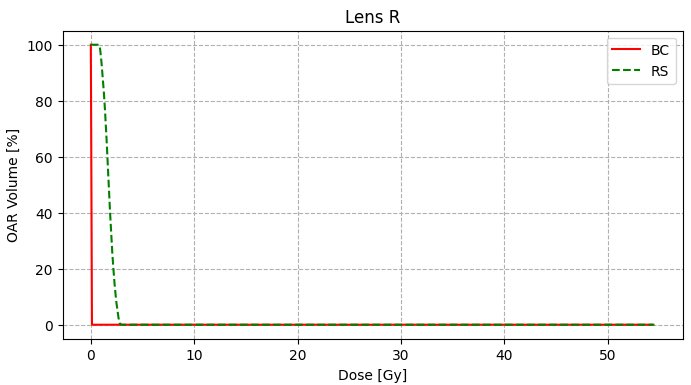


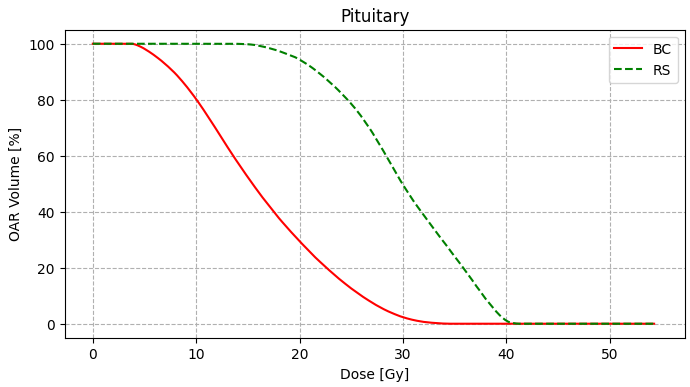


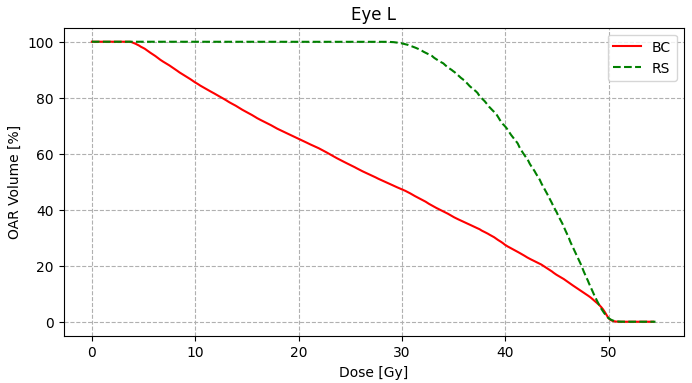


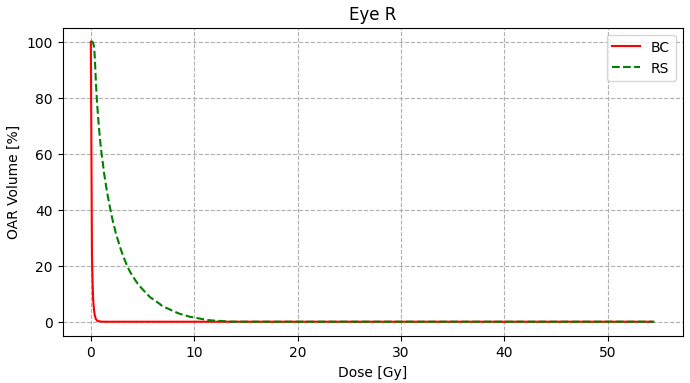


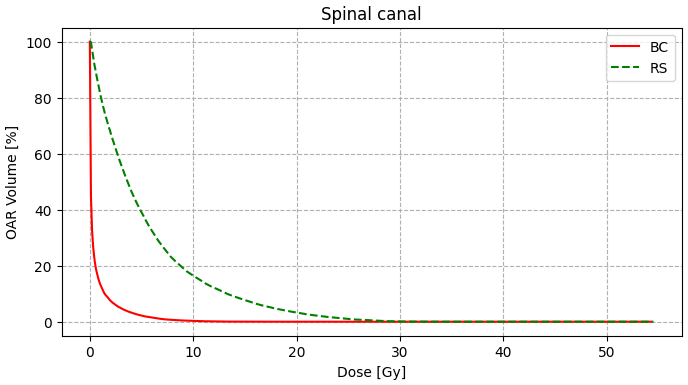


Case 6


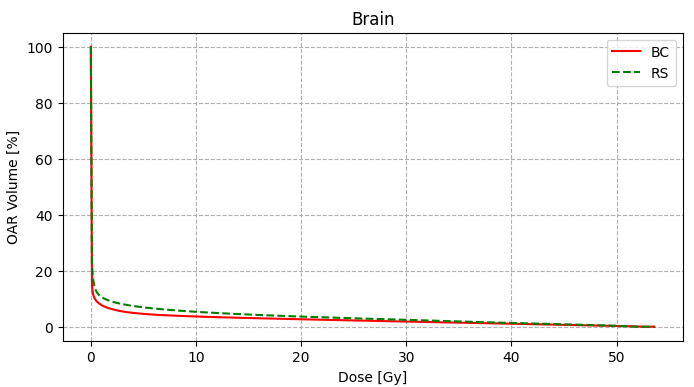


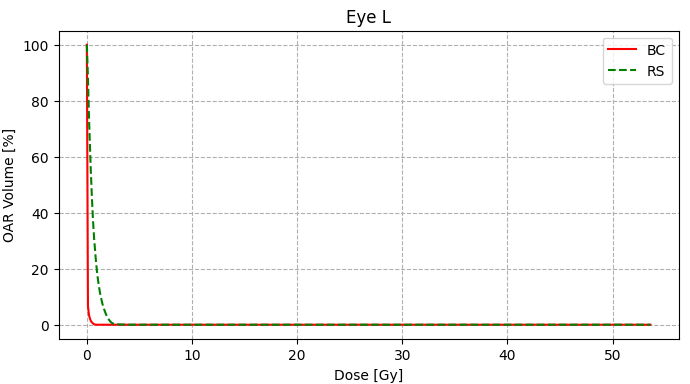


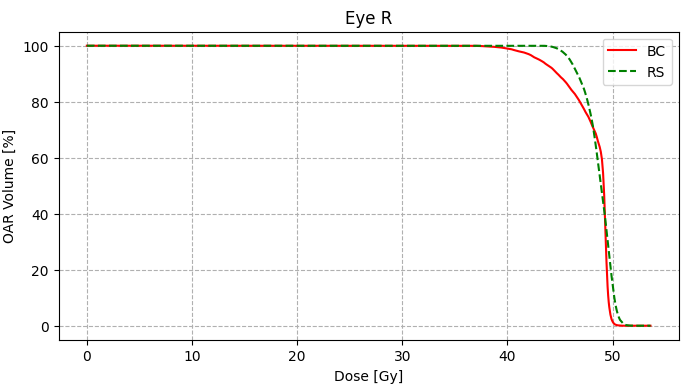


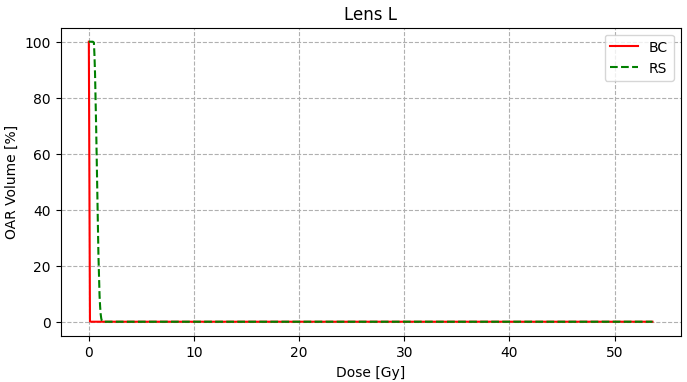


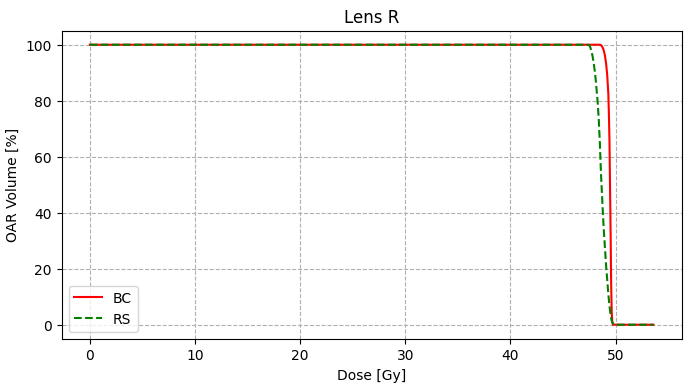


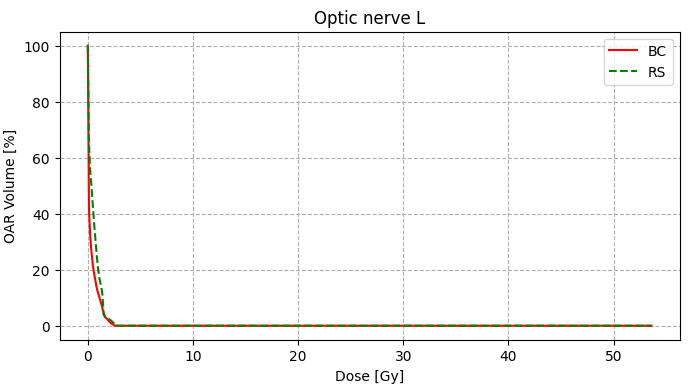


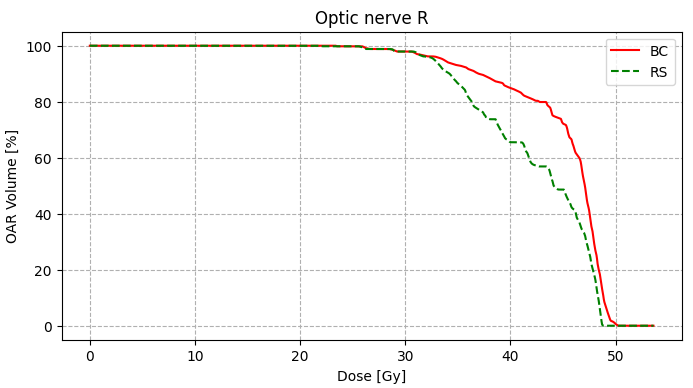


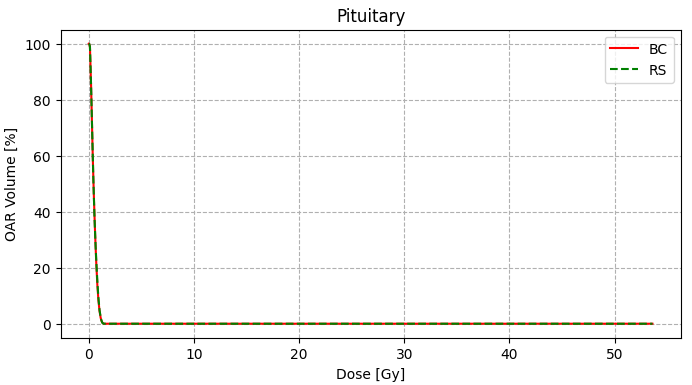


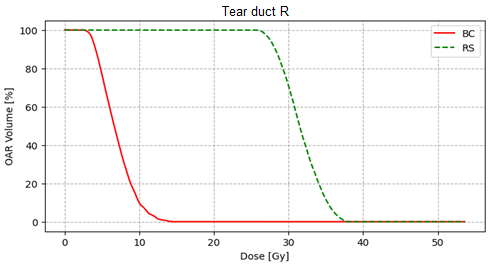

Supplement: Supplementary file 1 — Supplementary Material 1 [file 13014_2025_2646_MOESM1_ESM.docx]
