# Supplementary material for: Application of 3D-printed compensators for proton pencil beam scanning of shallowly localized pediatric tumors
Source: Radiat Oncol. 2025 Apr 29;20:66. doi: 10.1186/s13014-025-02646-3 (PMC12042327; doi:10.1186/s13014-025-02646-3)

**Application of 3D-printed compensators for proton pencil beam scanning of shallowly localized pediatric tumors**

**Agnieszka Wochnik^(1)^, Tomasz Kajdrowicz^(1)^, Gabriela Foltyńska^(1)^, Dawid Krzempek^(1)^, Katarzyna Krzempek^(1)^, Krzysztof Małecki^(2)^, Marzena Rydygier^(1)^, Jan Swakoń^(1)^, Paweł Olko^(1)^and Renata Kopeć^(1)^**

^1^Institute of Nuclear Physics Polish Academy of Sciences, Krakow, Poland

^2^University Children’s Hospital of Krakow, Poland

Corresponding author: [Agnieszka.Wochnik@ifj.edu.pl](mailto:Agnieszka.Wochnik@ifj.edu.pl)

Table S1. Characteristics of the selected patients and prescribed doses for the target areas.

|  | **Case 1** | **Case 2** | **Case 3** | **Case 4** | **Case 5** | **Case 6** |
| --- | --- | --- | --- | --- | --- | --- |
| **Gender** | male | male | male | male | female | male |
| **Tumor location** | upper left eyelid | left perioral area | right perioral area | left palate | left palate | right eye socket |
| **Tumor volume [cm^3^]** | 3.67 | 42.53 | 55.28 | 121.8 | 51.82 | 8.4 |
| **PTV1 [Gy_RBE_]** | 36 | 36 | 50.4 | 50.4 | 41.4 | 36 |
| **PTV2 [Gy_RBE_]** | 45 | 50.4 |  | 55.8 | 50.4 | 50.4 |
| **PTV3 [Gy_RBE_]** | 50.4 |  |  |  |  |  |

Dose distribution comparison

Case 1


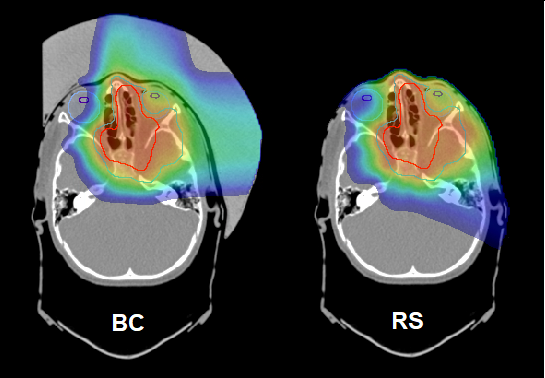


Case 2


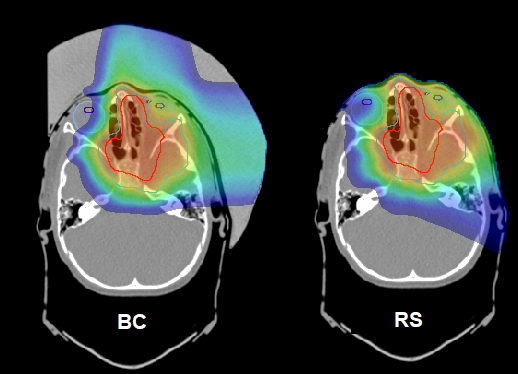


Case 3


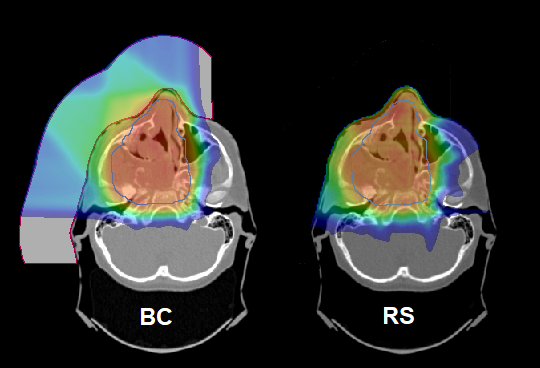


Case 4


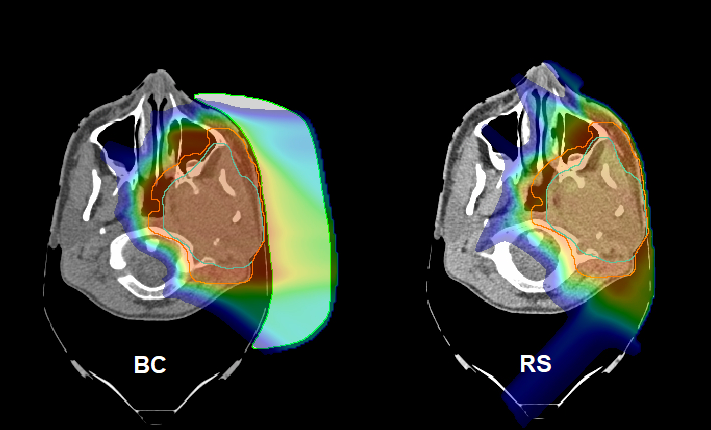


Case 5


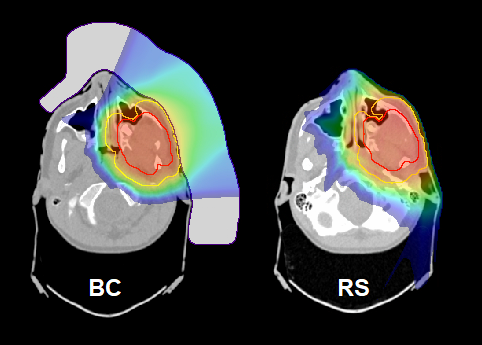


Case 6


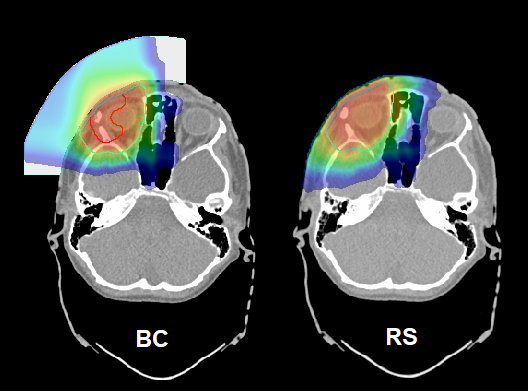


Dose-volume histograms for organs at risks. The comparison between the 3D printed beam compensator (BC) and range shifter (RS)

Case 1


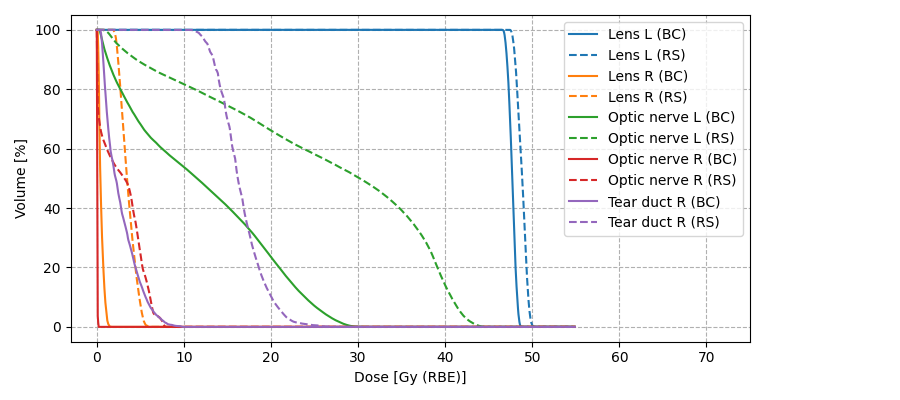


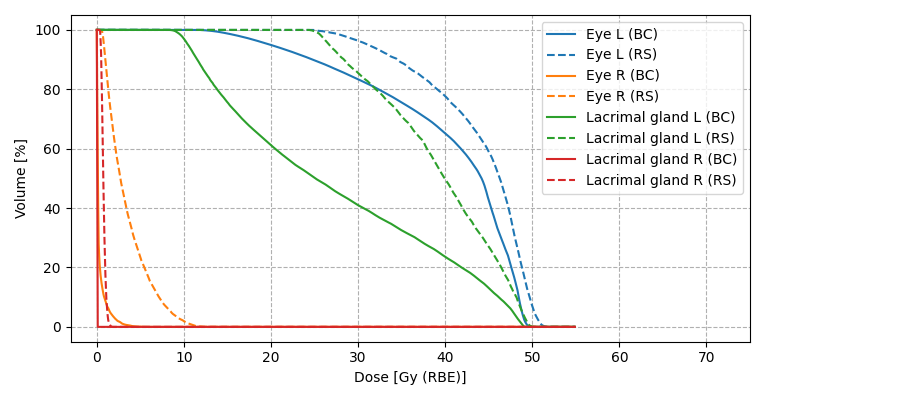


Case 2


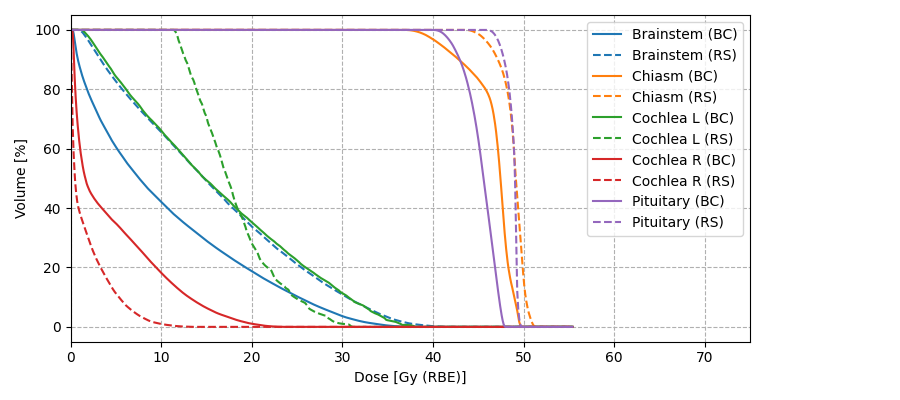


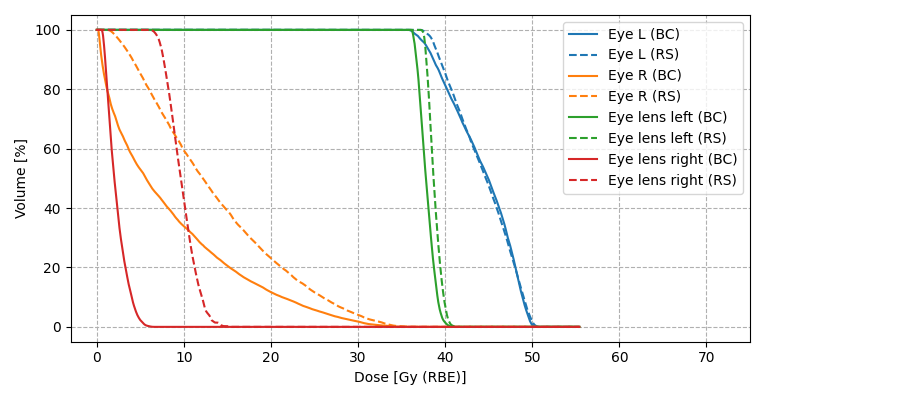


Case 3


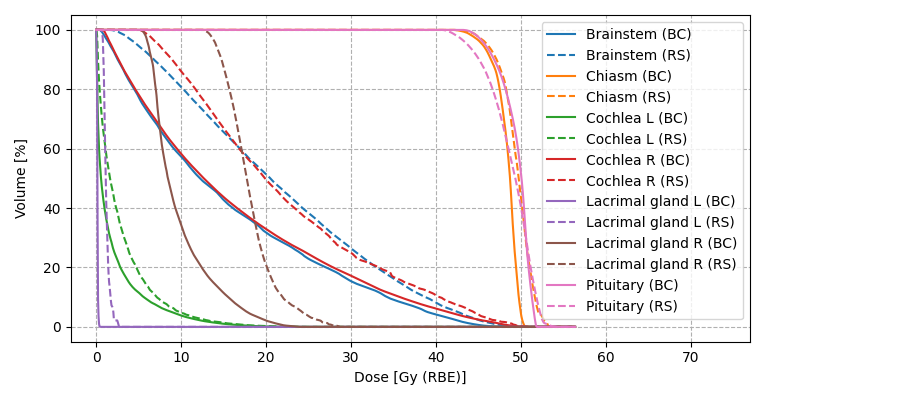


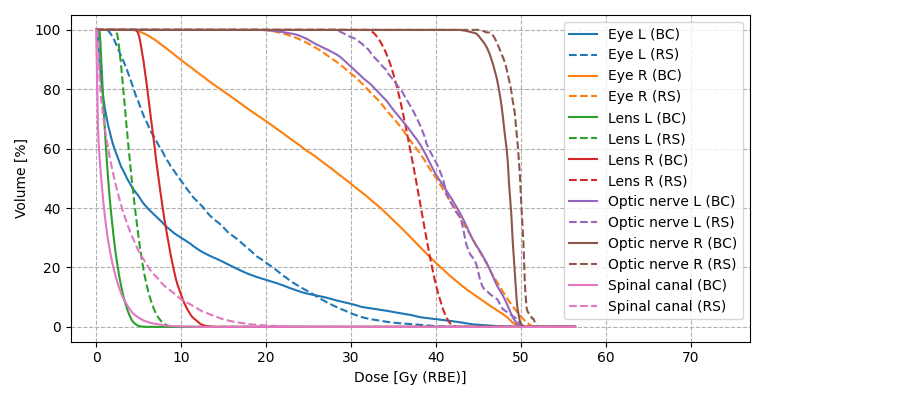


Case 4


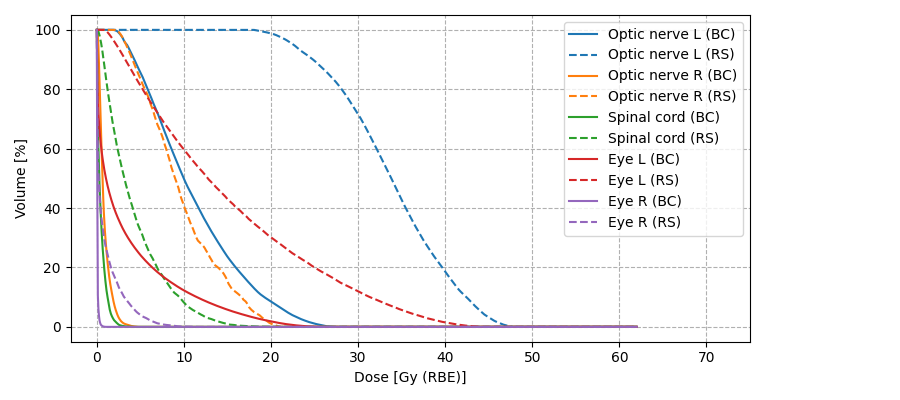


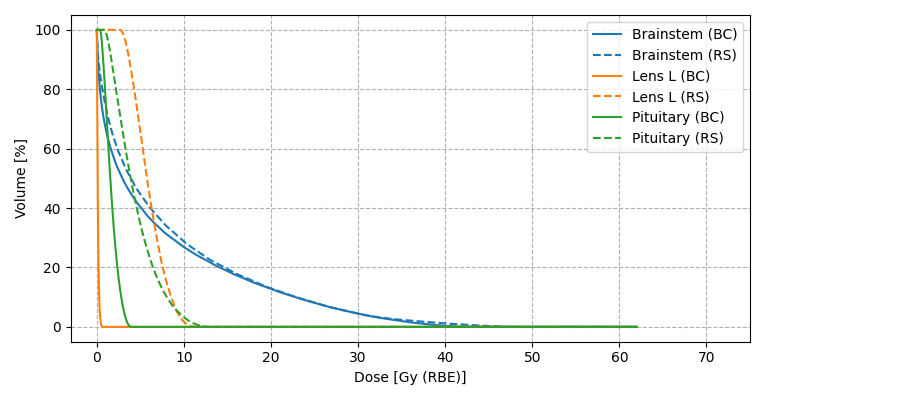


**Case 5**


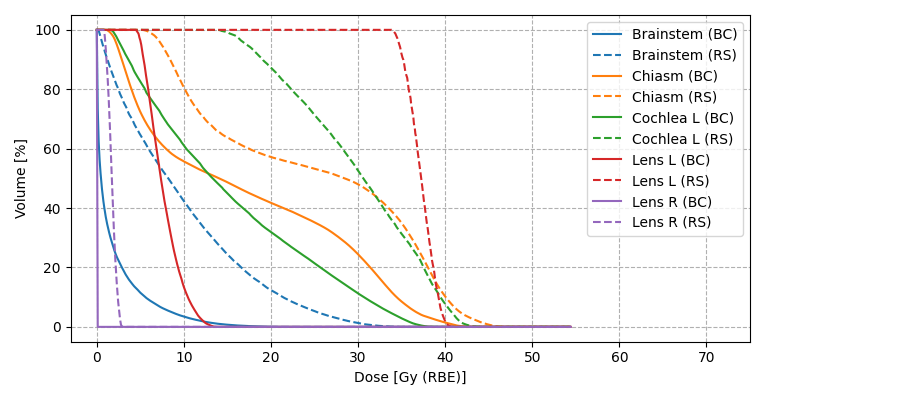


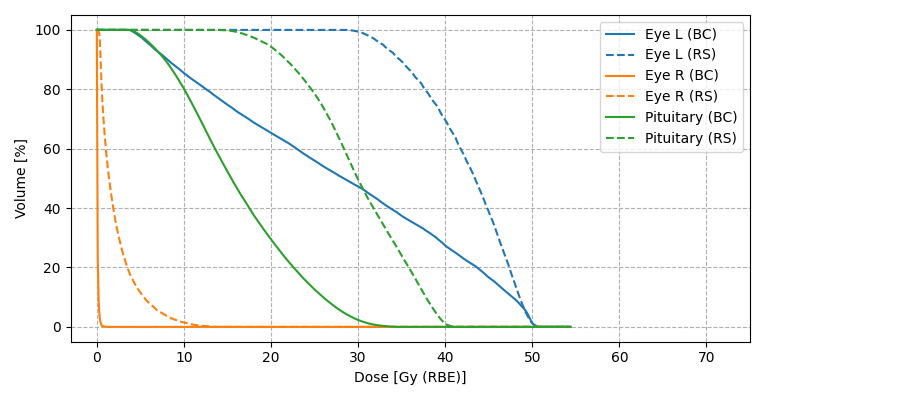


**Case 6**


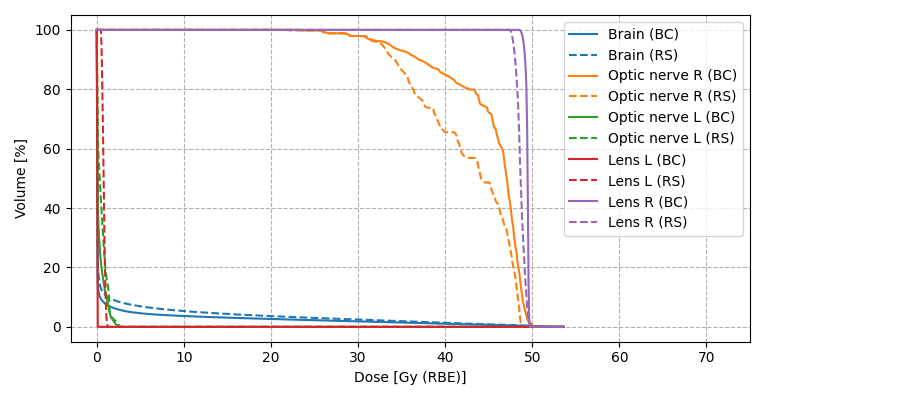


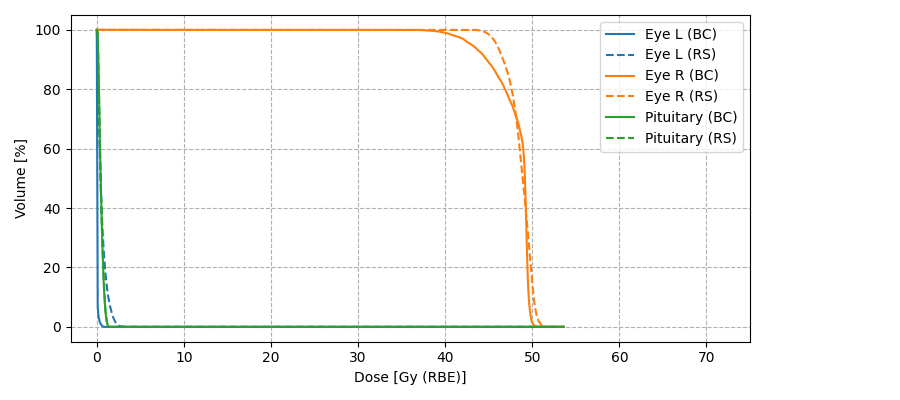

Supplement: Supplementary file 2 — Supplementary Material 2 [file 13014_2025_2646_MOESM2_ESM.docx]
