# Supplementary material for: Application of 3D-printed compensators for proton pencil beam scanning of shallowly localized pediatric tumors
Source: Radiat Oncol. 2025 Apr 29;20:66. doi: 10.1186/s13014-025-02646-3 (PMC12042327; doi:10.1186/s13014-025-02646-3)
Supplement: Supplementary file 3 — Supplementary Material 3 [file 13014_2025_2646_MOESM3_ESM.docx]

**Application of 3D-printed compensators for proton pencil beam scanning of shallowly localized pediatric tumors**

**Agnieszka Wochnik^(1)^, Tomasz Kajdrowicz^(1)^, Gabriela Foltyńska^(1)^, Dawid Krzempek^(1)^, Katarzyna Krzempek^(1)^, Krzysztof Małecki^(2)^, Marzena Rydygier^(1)^, Jan Swakoń^(1)^, Paweł Olko^(1)^and Renata Kopeć^(1)^**

^1^Institute of Nuclear Physics Polish Academy of Sciences, Krakow, Poland

^2^University Children’s Hospital of Krakow, Poland

Corresponding author: [Agnieszka.Wochnik@ifj.edu.pl](mailto:Agnieszka.Wochnik@ifj.edu.pl)

Table S1. Characteristics of the selected patients and prescribed doses for the target areas.

|  | **Case 1** | **Case 2** | **Case 3** | **Case 4** | **Case 5** | **Case 6** |
| --- | --- | --- | --- | --- | --- | --- |
| **Gender** | male | male | male | male | female | male |
| **Tumor location** | upper left eyelid | left perioral area | right perioral area | left palate | left palate | right eye socket |
| **Tumor volume [cm^3^]** | 3.67 | 42.53 | 55.28 | 121.8 | 51.82 | 8.4 |
| **PTV1 [Gy(RBE)]** | 36 | 36 | 50.4 | 50.4 | 41.4 | 36 |
| **PTV2 [Gy(RBE)]** | 45 | 50.4 |  | 55.8 | 50.4 | 50.4 |
| **PTV3 [Gy(RBE)]** | 50.4 |  |  |  |  |  |

Table S2. Hounsfield Unit (HU) limit values for accepting the homogeneity of the compensator. A red background indicates that the HU deviation is too high—the uncertainty of the calculated Relative Stopping Power (RSP) based on the calibration curve exceeds 3.5%.

|  |  | **the RSP uncertainty from the calibration curve** | | | | | |
| --- | --- | --- | --- | --- | --- | --- | --- |
|  |  | **SD HU** | | | | | |
|  |  | **±25** | **±50** | **±75** | **±80** | **±90** | **±100** |
| **mean HU** | **25** | **2.17%** | **4.35%** | **4.97%** | **5.21%** | **5.48%** | **7.05%** |
|  | **50** | **2.14%** | **3.89%** | **5.17%** | **5.38%** | **4.19%** | **6.74%** |
|  | **75** | **2.09%** | **4.17%** | **5.90%** | **6.16%** | **3.14%** | **7.16%** |
|  | **100** | **0.81%** | **2.67%** | **4.74%** | **7.46%** | **3.62%** | **6.46%** |
|  | **125** | **1.04%** | **2.13%** | **3.46%** | **3.87%** | **3.89%** | **5.51%** |
|  | **150** | **1.08%** | **2.16%** | **3.27%** | **3.50%** | **3.94%** | **4.45%** |
|  | **160** | **1.10%** | **2.15%** | **3.26%** | **3.49%** | **3.93%** | **4.39%** |
|  | **170** | **1.07%** | **2.16%** | **3.28%** | **3.49%** | **3.94%** | **4.39%** |
|  | **180** | **1.07%** | **2.17%** | **3.05%** | **3.50%** | **3.94%** | **4.38%** |
|  | **190** | **1.08%** | **2.18%** | **3.06%** | **3.50%** | **3.95%** | **4.38%** |
|  | **200** | **1.08%** | **2.18%** | **3.28%** | **3.50%** | **3.94%** | **4.29%** |
|  | **225** | **1.08%** | **2.17%** | **3.25%** | **3.47%** | **3.91%** | **4.35%** |
|  | **250** | **1.08%** | **2.15%** | **3.23%** | **3.44%** | **3.85%** | **4.30%** |

Dose distributions. The comparison between the 3D printed beam compensator (BC) and range shifter (RS).


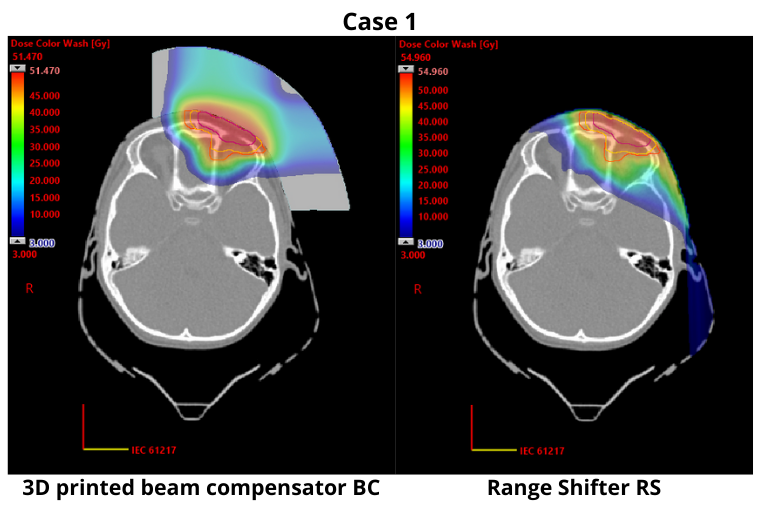


Figure S1. Dose distribution comparison between the 3D printed beam compensator (BC) and range shifter (RS) configurations for case 1. The red contour represents PTV1, the yellow contour represents PTV2, the purple contour represents PTV3.


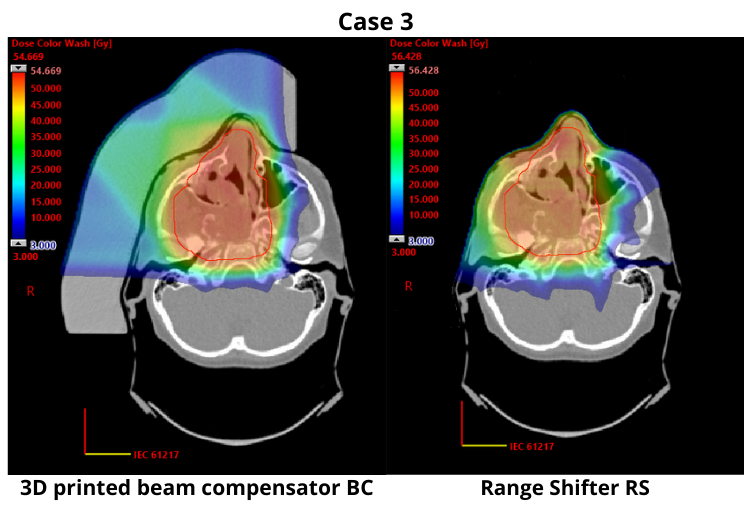


Figure S2. Dose distribution comparison between the 3D printed beam compensator (BC) and range shifter (RS) configurations for case 3. The red contour represents PTV1.


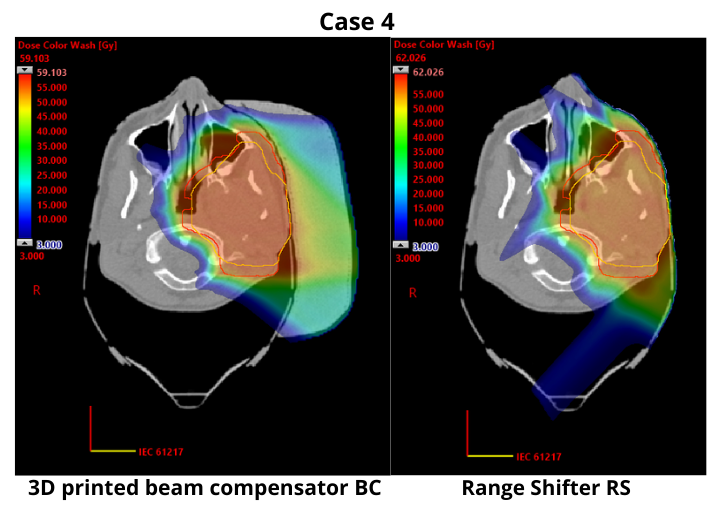


Figure S3. Dose distribution comparison between the 3D printed beam compensator (BC) and range shifter (RS) configurations for case 4. The red contour represents PTV1, while the yellow contour represents PTV2.


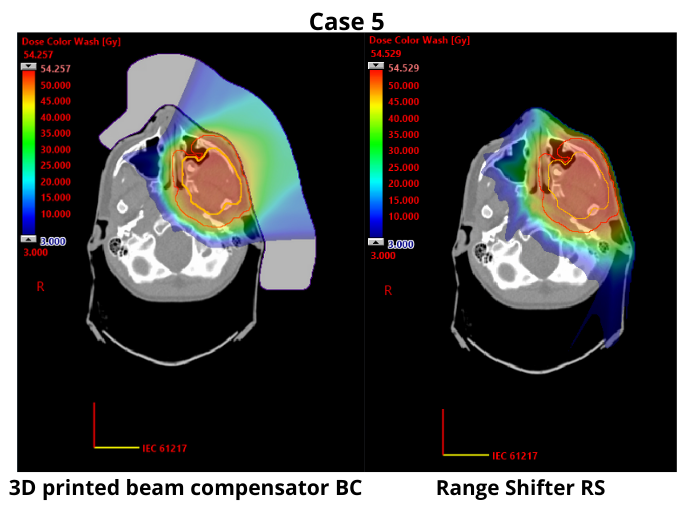


Figure S4. Dose distribution comparison between the 3D printed beam compensator (BC) and range shifter (RS) configurations for case 5. The red contour represents PTV1, while the yellow contour represents PTV2.


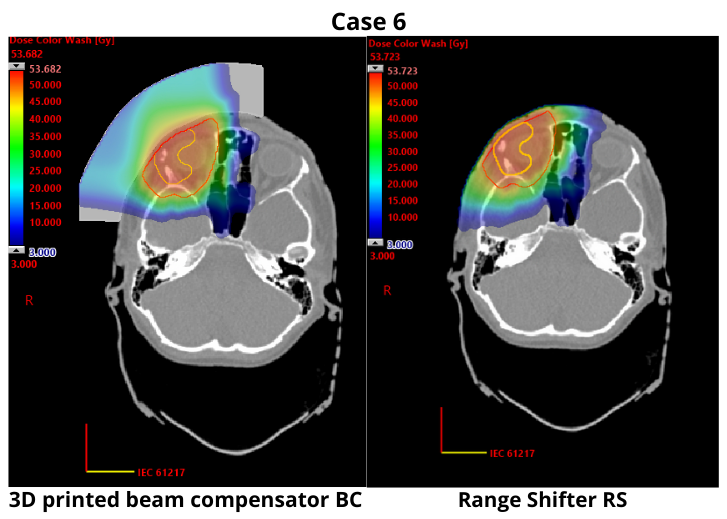


Figure S5. Dose distribution comparison between the 3D printed beam compensator (BC) and range shifter (RS) configurations for case 6. The red contour represents PTV1, while the yellow contour represents PTV2.

Dose-volume histograms (DVHs) for selected organs at risks. The comparison between the 3D printed beam compensator (BC) and range shifter (RS).

Case 1


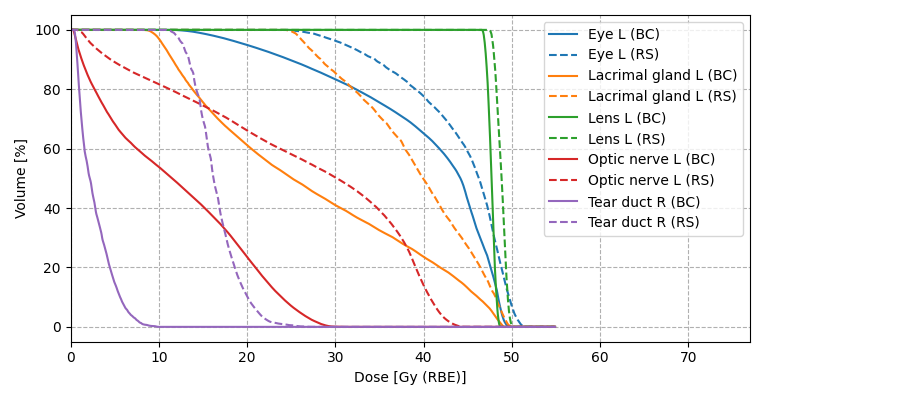


Figure S6. Dose-volume histograms (DVHs) for selected organs at risk for case 1. The comparison between the 3D printed beam compensator (BC) and range shifter (RS) configurations.

Case 2


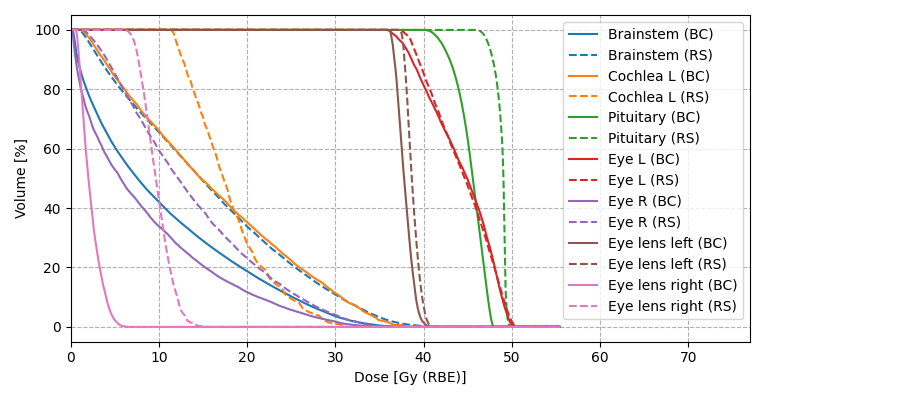


Figure S7. Dose-volume histograms (DVHs) for selected organs at risk for case 2. The comparison between the 3D printed beam compensator (BC) and range shifter (RS) configurations.

Case 3


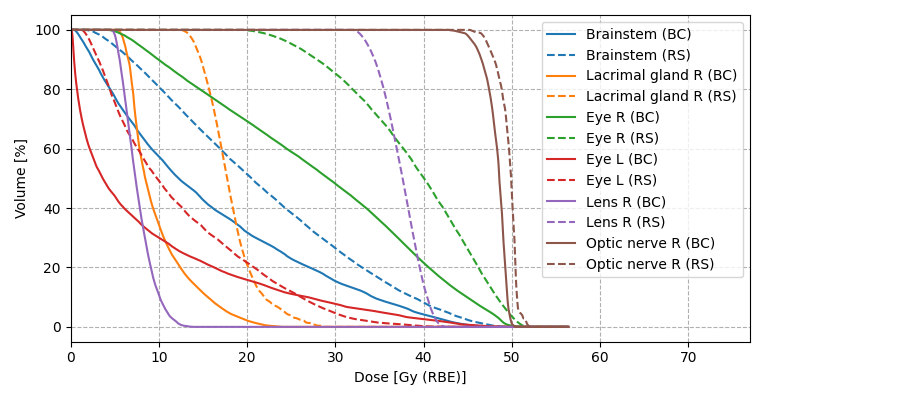


Figure S8. Dose-volume histograms (DVHs) for selected organs at risk for case 3. The comparison between the 3D printed beam compensator (BC) and range shifter (RS) configurations.

Case 4


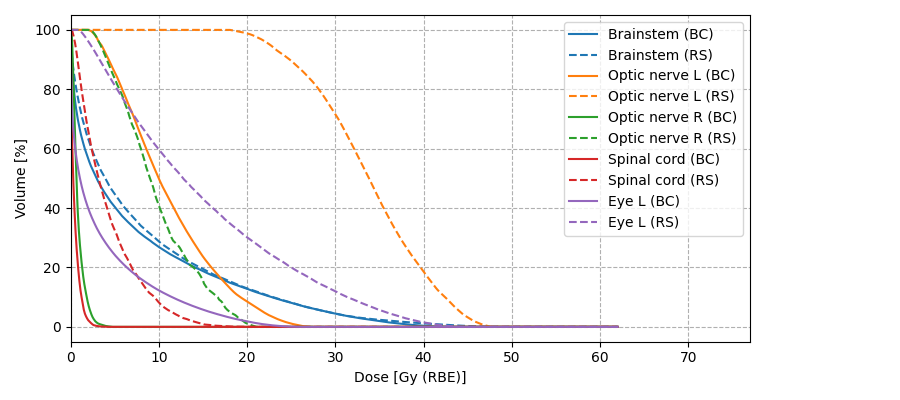


Figure S9. Dose-volume histograms (DVHs) for selected organs at risk for case 4. The comparison between the 3D printed beam compensator (BC) and range shifter (RS) configurations.

**Case 5**


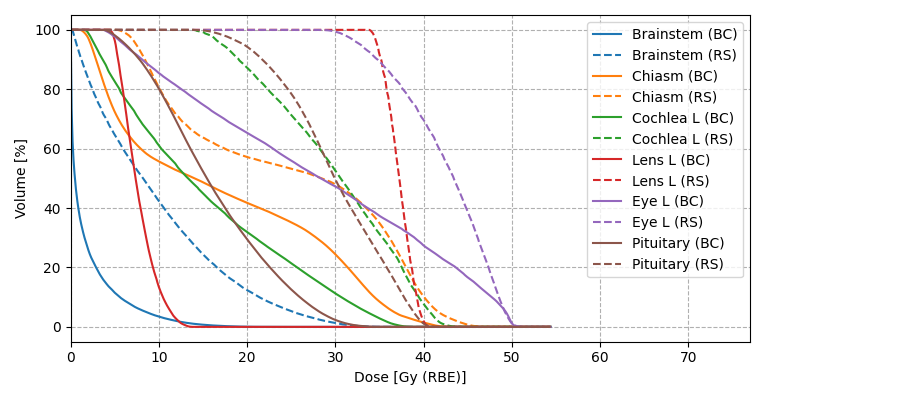


Figure S10. Dose-volume histograms (DVHs) for selected organs at risk for case 5. The comparison between the 3D printed beam compensator (BC) and range shifter (RS) configurations.

**Case 6**


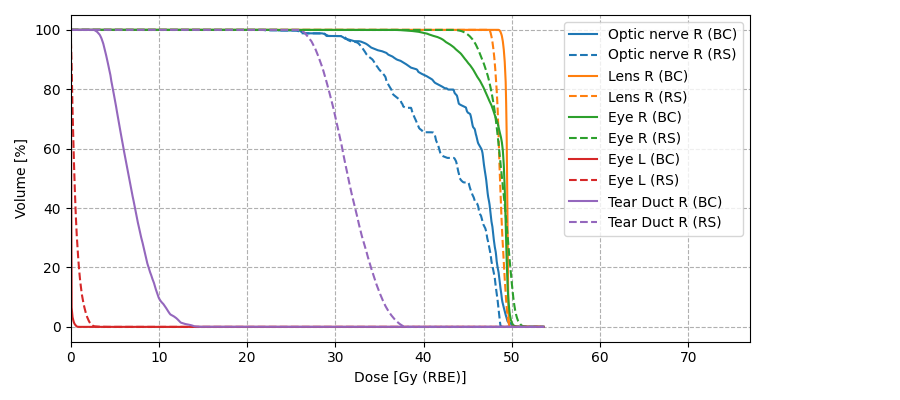


Figure S11. Dose-volume histograms (DVHs) for selected organs at risk for case 6. The comparison between the 3D printed beam compensator (BC) and range shifter (RS) configurations
